# Childhood Family Violence and Depressive Symptoms in Urban Chinese Women: The Moderating Role of Social Contact Frequency

**DOI:** 10.3390/bs16091698

**Published:** 2026-09-20

**Authors:** Shiyao Han, Li Zheng, Xiaohe Xu

**Affiliations:** 1 Department of Sociology and Psychology, School of Public Administration, Sichuan University, Chengdu 610207, China; 2024225010009@stu.scu.edu.cn; 2 Department of Sociology and Demography, University of Texas at San Antonio, San Antonio, TX 78249, USA; xiaohe.xu@utsa.edu

**Keywords:** childhood family violence, depressive symptoms, social contact frequency, stress-buffering hypothesis, ever-married women, urban China

## Abstract

Guided by the stress process model, this study examined whether childhood family violence—specifically, parental physical abuse (PPA) and witnessing interparental violence (WIPV)—was associated with depressive symptoms among ever-married women in Beijing and Chengdu, China, and whether these associations were moderated by social contact frequency. Drawing on data from the 2020 Family and Health Survey (*N* = 1849), we used ordinary least squares regression with heteroskedasticity-robust standard errors and conducted supplementary sensitivity analyses. In separate models, both PPA and WIPV were positively associated with depressive symptoms. Social contact frequency showed no significant main effect, but its interactions with both PPA and WIPV were significantly negative, a pattern consistent with the stress-buffering hypothesis. In combined models including both violence types, WIPV retained a significant unique association with depressive symptoms, whereas the PPA association was attenuated. The moderation findings suggest that social contact frequency may condition the violence–depression relationship, underscoring the potential importance of social relationships for responses to early-life adversity.

## 1. Introduction

The widely circulated expression, “spending a lifetime healing from childhood,” has gained considerable traction in contemporary Chinese public discourse, reflecting growing societal recognition of the enduring psychological consequences of adverse childhood experiences. Within this context, childhood family violence represents a form of early-life adversity with far-reaching implications for well-being across the life course. Its effects may accumulate over time, contributing to psychological vulnerability through mechanisms that are consistent with cumulative disadvantage theory (McLeod & Almazan, 2003; Dannefer, 2003; Ferraro et al., 2016; Felitti et al., 1998). A substantial body of research has demonstrated that exposure to parental physical abuse or witnessing interparental violence during childhood is associated with an elevated risk of depressive symptoms and disorders in adulthood (Mandelli et al., 2015; Kessler & Magee, 1993). Similar patterns have been documented in longitudinal studies conducted among Chinese populations (Lv & Li, 2023; Y. Li & Lu, 2020; Du et al., 2022).

Despite this growing evidence base, research in China has paid limited attention to distinguishing the long-term mental health consequences of distinct forms of childhood family violence, particularly witnessing interparental violence. As a result, the relative impact of direct versus indirect exposure to family violence remains insufficiently understood. Furthermore, the prevalence and consequences of childhood family violence may be systematically underestimated in the Chinese context, where corporal punishment and harsh parental discipline have often been embedded in culturally accepted child-rearing practices (J. Li & Cao, 2025; Jiang & Jiang, 2022). Empirical studies in Chinese settings indicate that corporal punishment, psychological aggression, and more severe physical abuse may coexist within families, and that adolescents’ perceived normality of harsh discipline may shape their recognition and reporting of harmful experiences (Tang, 2006; Wang & Liu, 2014; Liu & Wang, 2018). Such cultural norms may obscure the boundary between discipline and abuse, potentially reducing the likelihood that childhood violence is recognized or retrospectively reported as maltreatment. Consequently, a more nuanced examination of specific forms of childhood family violence is needed to advance understanding of their differential associations with adult mental health outcomes.

Guided by the stress process model, this study investigates the long-term associations between childhood family violence and depressive symptoms among ever-married women in Beijing and Chengdu using data from the 2020 Family and Health Survey. The focus on ever-married women reflects the design of the survey, which collected detailed information on marital, family, childhood, and social experiences among women with marital histories. This focus allows the study to examine depressive symptoms within adult family and social contexts, while limiting the generalizability of findings beyond ever-married women in these urban settings. Specifically, the study examines whether social contact frequency, conceptualized as a structural dimension of social embeddedness, moderates the observed associations between childhood family violence and depressive symptoms. In doing so, the study makes two primary contributions to the literature. First, it differentiates two distinct forms of childhood family violence—parental physical abuse and witnessing interparental violence—within an urban Chinese context where these experiences have often been examined collectively. Second, it examines whether routine face-to-face contact with family members and friends and participation in social gatherings are associated with weaker violence–depression associations. Because the data are cross-sectional and retrospective, these moderation findings are interpreted as conditional associations rather than evidence that social contact causally protects women from the long-term consequences of early-life violence exposure.

## 2. Literature Review

### 2.1. Childhood Family Violence and Adult Depressive Symptoms

Family violence is broadly defined as acts of physical, psychological, or sexual harm occurring among family members, encompassing both intimate partner violence and intergenerational forms of maltreatment (Huang, 2002). The present study focuses on two specific forms of childhood exposure to family violence: parental physical abuse (PPA), which involves direct infliction of physical harm on a child by a parent, and witnessing interparental violence (WIPV), which refers to a child’s exposure to violent interactions between parents. Although WIPV does not entail direct physical victimization, it represents a significant source of psychological stress and insecurity during childhood and has been identified as a serious threat to healthy emotional and psychological development (Huang, 2002; Greenfield & Marks, 2010a). By exposing children to a chronic environment of fear, conflict, and instability, both PPA and WIPV may have enduring consequences for mental health and well-being across the life course.

A substantial body of international research has documented the detrimental mental health consequences of childhood exposure to family violence. Meta-analytic evidence indicates that parental physical abuse is associated with a wide range of negative developmental and psychological outcomes, including depression, anxiety, aggression, and antisocial behavior (Gershoff, 2002). Likewise, children who witness interparental violence exhibit levels of emotional, behavioral, and social maladjustment comparable to those observed among children who experienced direct physical victimization (Kitzmann et al., 2003). Longitudinal research further demonstrates that exposure to both parental physical abuse and witnessing interparental violence increases the risk of depressive symptoms and depressive disorders in adulthood, suggesting that the psychological effects of childhood family violence extend well beyond the immediate developmental period (Mandelli et al., 2015; Shaw & Krause, 2002). Collectively, these findings underscore the enduring impact of both direct and indirect forms of childhood family violence on mental health across the life course.

Evidence from the Chinese context remains comparatively limited and has focused predominantly on parental physical abuse. Existing studies consistently report positive associations between childhood physical abuse and depressive symptoms in adulthood (Y. Li & Lu, 2020; Du et al., 2022; Lv & Li, 2023). In contrast, considerably less attention has been devoted to the long-term mental health consequences of witnessing interparental violence, despite evidence suggesting that its psychological impact may be comparable to that of direct victimization (Kitzmann et al., 2003). This omission represents an important gap in the literature, particularly given that parental physical abuse and witnessing interparental violence often co-occur within the same family environment (Felitti et al., 1998). Although related, these experiences reflect distinct dimensions of childhood family violence, involving direct and indirect forms of exposure to violence, respectively. Examining both forms simultaneously may therefore provide a more comprehensive understanding of the lasting mental health implications of childhood family violence among Chinese women and help clarify whether different forms of exposure exert differential effects on depressive symptoms in adulthood.

### 2.2. Life Course Theory and the Stress Process Model

Two complementary theoretical perspectives have been advanced to explain the long-term associations between childhood family violence and adult mental health. The first, life course theory, emphasizes the enduring influence of early-life experiences on subsequent developmental trajectories and later-life outcomes. Childhood occupies a critical position in the life course, serving as a formative period for core aspects of self-concept, emotional regulation, and social functioning (McLeod & Almazan, 2003). From this perspective, mental health disparities observed in adulthood are understood, at least in part, as the cumulative manifestation of advantages and disadvantages that originate early in life and unfold over time (Dannefer, 2003; Ferraro et al., 2016). Exposure to family violence during childhood represents a significant source of early adversity that may disrupt normative emotional and social development, thereby increasing susceptibility to psychological distress in later life. Through processes of cumulative disadvantage, the effects of such traumatic experiences may persist and even intensify over time, contributing to enduring patterns of mental health vulnerability across the life course.

The stress process model offers a complementary perspective by emphasizing the dynamic interplay between stressors and resources in shaping mental health outcomes (Pearlin, 1989, 2010). Within this framework, childhood family violence can be conceptualized as an early-life stressor that may be associated with later psychological well-being. Exposure to violence during childhood may generate immediate distress and may also be linked to later vulnerability to stress and reduced access to coping resources (Sperry & Widom, 2013). However, the present study does not directly measure intervening stressors, coping processes, or changes in resources over time. Concepts such as stress proliferation and resource erosion are used here as interpretive frameworks rather than as mechanisms directly tested in the empirical analysis. This perspective is consistent with the stress-depression framework, which posits that depressive symptoms are shaped by the accumulation and interaction of stressors encountered across different stages of life (Hammen, 2005).

Compared with life course theory, which emphasizes the cumulative impact of early advantages and disadvantages across the life course, the stress process model focuses more explicitly on how exposure to stressors and access to coping resources may jointly shape mental health trajectories (Greenfield & Marks, 2010b). Together, these perspectives suggest that childhood family violence may be associated with depressive symptoms in adulthood is consistent with both cumulative disadvantage and stress-process perspectives. Within this framework, both parental physical abuse and witnessing interparental violence are conceptualized as early stressors that may be linked to psychological vulnerability in adulthood.

**Hypothesis 1a.**  *Parental physical abuse (PPA) is positively associated with depressive symptoms among ever-married women in urban China.*

**Hypothesis 1b.**  *Witnessing interparental violence (WIPV) is positively associated with depressive symptoms among ever-married women in urban China.*

### 2.3. Social Contact Frequency and the Stress-Buffering Hypothesis

Social relationships have long been linked to mental health, but the literature distinguishes among social networks, social integration, social support, and social contact (Cohen, 2004). Structural dimensions of social relationships capture patterns of social ties and interactions, whereas functional social support refers to the emotional, informational, and instrumental resources that may be available through those ties (House et al., 1988; Cohen & Wills, 1985; Berkman et al., 2000; Umberson & Montez, 2010). This distinction is important for the present study because the available survey items measure routine face-to-face contact and social participation rather than relationship quality, perceived support, or received support.

From the perspective of the stress process model, social ties may matter for mental health because they provide opportunities for social engagement and access to supportive and other social resources (Lin et al., 1999; Kawachi & Berkman, 2001). At the same time, more frequent contact does not necessarily imply supportive or safe relationships. This point is particularly relevant for respondents exposed to family violence, for whom family contact may involve ambivalent or potentially stressful ties rather than uniformly protective resources (Holt-Lunstad & Uchino, 2019; Offer, 2020; Lee & Szinovacz, 2016). Accordingly, the present study uses the more precise term social contact frequency to capture the frequency of face-to-face interactions with family members and friends and participation in social gatherings, reflecting opportunities for interpersonal engagement rather than the quality or function of these relationships.

The stress-buffering hypothesis posits that social resources may be most consequential under conditions of elevated stress (Cohen & Wills, 1985; Kawachi & Berkman, 2001). Consistent with this framework, prior research suggests that social relationships and social support may weaken associations between childhood adversity and later-life mental health problems (Cheong et al., 2017; Sperry & Widom, 2013). Other studies show that in-person contact with family and friends is associated with depression risk, although contact frequency and support quality are not interchangeable (Teo et al., 2013, 2015; Cornwell & Waite, 2009). These findings provide a rationale for examining whether social contact frequency moderates the observed association between childhood family violence and depressive symptoms.

Building on this theoretical framework, the present study examines whether social contact frequency is associated with depressive symptoms and whether it moderates the observed associations between PPA/WIPV and depressive symptoms among ever-married women in urban China.

**Hypothesis 2.** 
*Social contact frequency is negatively associated with depressive symptoms among ever-married women in urban China.*

**Hypothesis 3a.** 
*Social contact frequency attenuates the positive association between parental physical abuse and depressive symptoms among ever-married women in urban China.*

**Hypothesis 3b.** 
*Social contact frequency attenuates the positive association between witnessing interparental violence and depressive symptoms among ever-married women in urban China.*

## 3. Methods

### 3.1. Study Population and Data Source

This study draws on data from the 2020 Family and Health Survey conducted between September and November 2020 in Beijing and Chengdu, China. The survey employed a multistage random sampling design to collect information from ever-married women residing in these two urban centers. The analytic focus on ever-married women reflects the design of the survey, which was structured around women’s marital, family, childhood, and social relationship experiences. In each city, residential communities were randomly selected, households were then sampled using systematic procedures, and one eligible ever-married woman who had lived in the city for at least six months was selected from each household. A total of 2086 ever-married women aged 20–99 were interviewed, including 1265 respondents from Beijing and 821 respondents from Chengdu. Trained interviewers conducted face-to-face surveys, and all respondents completed the questionnaires independently without interference from other household members. The available survey documentation did not provide the denominator required to calculate a response rate. The survey protocol received approval from the Ethics Committee of a university-affiliated hospital ethics committee in China (Approval No. K2019032). All participants provided informed consent before participation and received modest compensation.

The survey includes detailed measures of childhood family experiences, family and social relationships, and physical and mental health. Following the analytic sample definition used for this study, respondents older than 75 were excluded (*n* = 18), and cases with missing values on the focal variables or covariates were removed (*n* = 219), yielding a final analytic sample of 1849 ever-married women. Variable-specific missingness, the sample-selection flow, and comparisons between included and excluded cases are reported in Supplementary Table S1. Responses coded as “not applicable” on individual social contact items were treated as structurally missing because they indicated that the corresponding relationship or activity did not apply to the respondent, rather than ordinary item nonresponse. When respondents had valid responses on other social contact items, the social contact frequency score was calculated using the available valid items. Missingness on variables other than PPA, WIPV, and the social contact items was minimal: only one respondent had missing hukou status, and no missing values occurred on the other focal variables or covariates (Supplementary Table S1). Multiple imputation was not used because missingness was concentrated primarily in the retrospective family-violence items and structurally “not applicable” social-contact responses, with the latter not representing ordinary item nonresponse. Cases with missing values required for the regression models were therefore handled using listwise deletion. Sampling weights, cluster identifiers, and strata variables were not available in the analytic dataset; therefore, survey-adjusted models could not be estimated.

### 3.2. Measures

#### 3.2.1. Depressive Symptoms

Depressive symptoms were measured using the 9-item Patient Health Questionnaire (PHQ-9), a widely used screening instrument for depressive symptomatology that has been validated in Chinese community samples (Bian et al., 2009; Kroenke et al., 2001). Respondents reported the frequency with which they experienced each symptom during the previous two weeks on a four-point scale ranging from 0 (not at all) to 3 (nearly every day). Item responses were summed to generate a total PHQ-9 score, with higher scores indicating greater levels of depressive symptoms. Consistent with prior research, the total score was treated as a continuous measure in the analyses. The Cronbach’s alpha coefficient for the PHQ-9 was 0.875.

#### 3.2.2. Childhood Family Violence

PPA and WIPV were measured using retrospective items based on questions previously used by Xu et al. (2019) to assess direct and witnessed exposure to family violence during childhood. For the present survey, the items were translated into Chinese, reviewed for linguistic and cultural appropriateness, and refined following pilot testing before field administration. The questionnaire introduced this section by asking respondents to recall experiences while growing up; it did not specify a precise age cutoff such as age 18. WIPV was measured with two items asking how often respondents saw their father hit their mother and how often they saw their mother hit their father. PPA was measured with two parallel items asking how often respondents were hit by their father and by their mother, with examples including being spanked or slapped. These measures are therefore interpreted as retrospective reports of the frequency of specific parental behaviors rather than as validated diagnostic measures of childhood abuse. Response options were 1 = very frequently, 2 = frequently, 3 = sometimes, 4 = rarely, 5 = never, and 6 = not applicable. Responses were reverse-coded to range from 0 to 4, with higher scores indicating more frequent exposure; “not applicable” responses were treated as missing. For both PPA and WIPV, the primary measure was calculated as the mean of the two constituent items. Item-level distributions are reported in Supplementary Table S2. The two items comprising PPA were strongly correlated (r = 0.73, *p* < 0.001), as were the two items comprising WIPV (r = 0.77, *p* < 0.001), indicating substantial within-measure correspondence; however, these correlations do not establish construct validity. Because averaging father- and mother-related items may dilute severe exposure from one parent or one direction of interparental violence, supplementary analyses used maximum exposure scores for PPA and WIPV.

#### 3.2.3. Social Contact Frequency

Social contact frequency was measured using seven items from the social participation section of the survey. Six items assessed the frequency of reciprocal in-person visits with parents, siblings, and friends (Q186–Q191), and one item measured participation in formal or informal gatherings outside the family (Q192). Items were reverse-coded to range from 0 (never) to 4 (very frequently), with higher values indicating more frequent social contact. Responses of “not applicable” were treated as missing rather than as low contact. The composite score was calculated as the mean of available valid items; the distribution of the number of valid items is reported in Supplementary Table S1. The seven-item composite demonstrated high internal consistency (Cronbach’s α = 0.887). Because this measure captures contact frequency rather than relationship quality or perceived/received support, supplementary analyses distinguished family contact, friend contact, and social participation domains.

#### 3.2.4. Control Variables

All models adjusted for a set of sociodemographic and health-related covariates, including age (continuous and mean-centered), years of education (continuous and mean-centered), log monthly household income (continuous and centered), marital status (1 = currently married or remarried), self-rated health (measured on a 1–7 scale), household registration status (1 = urban hukou), ethnicity (1 = ethnic minority), political affiliation (1 = Chinese Communist Party [CCP] member), and city of residence (1 = Chengdu, 0 = Beijing). These variables were treated as covariates rather than definitive confounders because several, including education, household income, marital status, political affiliation, and self-rated health, may have been shaped after childhood exposure. Because self-rated health may be a post-exposure variable rather than a conventional confounder, sensitivity analyses excluding self-rated health were also conducted. Additional sensitivity analyses further adjusted for paternal and maternal education when the respondent was age 12 as indicators of childhood socioeconomic conditions. Because these measures contained additional missing values, these models included 1732 respondents.

### 3.3. Statistical Analysis Plan

All analyses were conducted using Stata 18.0. OLS regression was employed as the primary modeling approach for interpretability. A series of regression models was estimated. Model 1 included the control variables and social contact frequency. Models 2 and 3 separately introduced PPA and WIPV to assess their total associations with depressive symptoms. Models 4 and 5 added the interaction terms between social contact frequency and each violence measure to test the moderation hypotheses. Interaction terms were constructed after standardizing the focal violence variables and social contact frequency. Specifically, Model 1 assessed Hypothesis 2, Model 2 assessed Hypothesis 1a, Model 3 assessed Hypothesis 1b, and Models 4 and 5 assessed Hypotheses 3a and 3b, respectively. All models were estimated using heteroskedasticity-robust standard errors to account for potential violations of homoscedasticity assumptions.

To evaluate the unique main-effect associations of PPA and WIPV, Model 6 included both exposures simultaneously. Models 7 and 8 then added the interaction terms separately: Model 7 included PPA × social contact frequency, and Model 8 included WIPV × social contact frequency; both models retained the main effects of PPA, WIPV, and social contact frequency. This split-interaction specification avoided estimating two interaction terms from highly correlated violence measures in the same equation. Multicollinearity was assessed using variance inflation factors and the coefficient correlation matrix. We also conducted an additional joint-exposure analysis that classified respondents into no exposure, PPA only, WIPV only, and co-exposure groups. Significant interactions were probed using simple slopes at the 25th, 50th, and 75th percentiles of social contact frequency and Johnson–Neyman-style plots within the observed range of the moderator.

Robustness checks included maximum-exposure scoring for PPA/WIPV, domain-specific social contact measures, models excluding self-rated health, influence diagnostics, Poisson and negative binomial models, and logistic models using PHQ-9 cutoffs of 5 and 10. These PHQ-9 cutoff models were treated as sensitivity analyses of elevated depressive symptom burden rather than as clinical diagnoses. The combined models are interpreted as supplementary comparative analyses rather than as primary tests of the original hypotheses; the split-interaction models are not interpreted as tests of statistically independent moderation effects.

## 4. Results

### 4.1. Descriptive Statistics and Correlation Analysis

Table 1 presents the descriptive statistics for all focal and control variables examined in this study. The sample had a mean PHQ-9 score of 3.52 (SD = 3.76). The mean scores for WIPV and PPA were 0.48 (SD = 0.87) and 0.70 (SD = 0.90), respectively, on scales ranging from 0 to 4. In the analytic sample, 52.2% reported any PPA and 35.7% reported any WIPV; 8.4% and 6.7%, respectively, reported frequent or very frequent exposure by at least one parent or in at least one direction. The joint-exposure distribution was 44.6% neither exposure, 19.7% PPA only, 3.2% WIPV only, and 32.4% both. The mean social contact frequency score was 1.92 (SD = 0.80), reflecting routine in-person contact with family members and friends and participation in social gatherings. Participants had a mean age of 47.05 years and approximately 12 years of education; most were currently married and held urban hukou status.

Table 2 presents correlations among the focal study variables. Both forms of childhood family violence were positively associated with depressive symptoms. Greater exposure to WIPV and PPA was associated with higher levels of depressive symptoms (r = 0.300, *p* < 0.001; r = 0.248, *p* < 0.001, respectively). Social contact frequency showed a small negative correlation with depressive symptoms (r = −0.046, *p* < 0.05). WIPV and PPA were strongly correlated (r = 0.762, *p* < 0.001), indicating substantial overlap between direct and witnessed forms of childhood family violence. Social contact frequency was positively correlated with both WIPV and PPA, although these associations were relatively small (r = 0.153 and 0.128, respectively; *p* < 0.001). These bivariate associations are descriptive and should not be interpreted as causal evidence.

### 4.2. Main Effects of Childhood Family Violence and Social Contact Frequency

Table 3 presents OLS regression models with heteroskedasticity-robust standard errors examining the associations of childhood family violence and social contact frequency with depressive symptoms. Model 1 includes control variables and social contact frequency as the baseline specification. In Model 2, PPA was positively associated with depressive symptoms (b = 0.585, *p* < 0.001), after adjusting for all control variables and social contact frequency, supporting Hypothesis 1a. Model 3 examined WIPV, which was also significantly and positively associated with depressive symptoms (b = 0.795, *p* < 0.001), providing support for Hypothesis 1b. The inclusion of childhood family violence indicators increased the explained variance in depressive symptoms, with R^2^ increasing from 0.135 in the baseline model to 0.154 and 0.166 in the PPA and WIPV models, respectively.

Across Models 1–3, the coefficients for social contact frequency were not statistically significant, providing no support for Hypothesis 2. The moderation hypotheses were tested separately in Models 4 and 5.

### 4.3. Moderating Role of Social Contact Frequency

Table 4 displays the moderation models testing Hypothesis 3 using heteroskedasticity-robust standard errors. As shown in Model 4, the interaction between PPA and social contact frequency was statistically significant and negative (b = −0.442, *p* < 0.001), indicating that the positive association between PPA and depressive symptoms was weaker at higher levels of social contact frequency. This finding provides support for Hypothesis 3a.

Model 5 shows that the interaction term between WIPV and social contact frequency was also significant and negative (b = −0.487, *p* < 0.001), indicating that the positive association between WIPV and depressive symptoms was weaker at higher levels of social contact frequency. Thus, Hypothesis 3b was supported in the separate WIPV model.

Across both models, the main effect of social contact frequency was not statistically significant. In these interaction models, the coefficient for social contact frequency represents its association with depressive symptoms when the standardized violence variable equals zero (i.e., its sample mean), rather than a universal association between contact and depressive symptoms for all respondents. The interaction results are consistent with a stress-buffering pattern, indicating that social contact frequency attenuates the positive associations between childhood family violence and depressive symptoms. However, because the data are cross-sectional, they should be interpreted as statistical moderation in observed associations rather than as evidence of causal protection.

**Figure 1:**
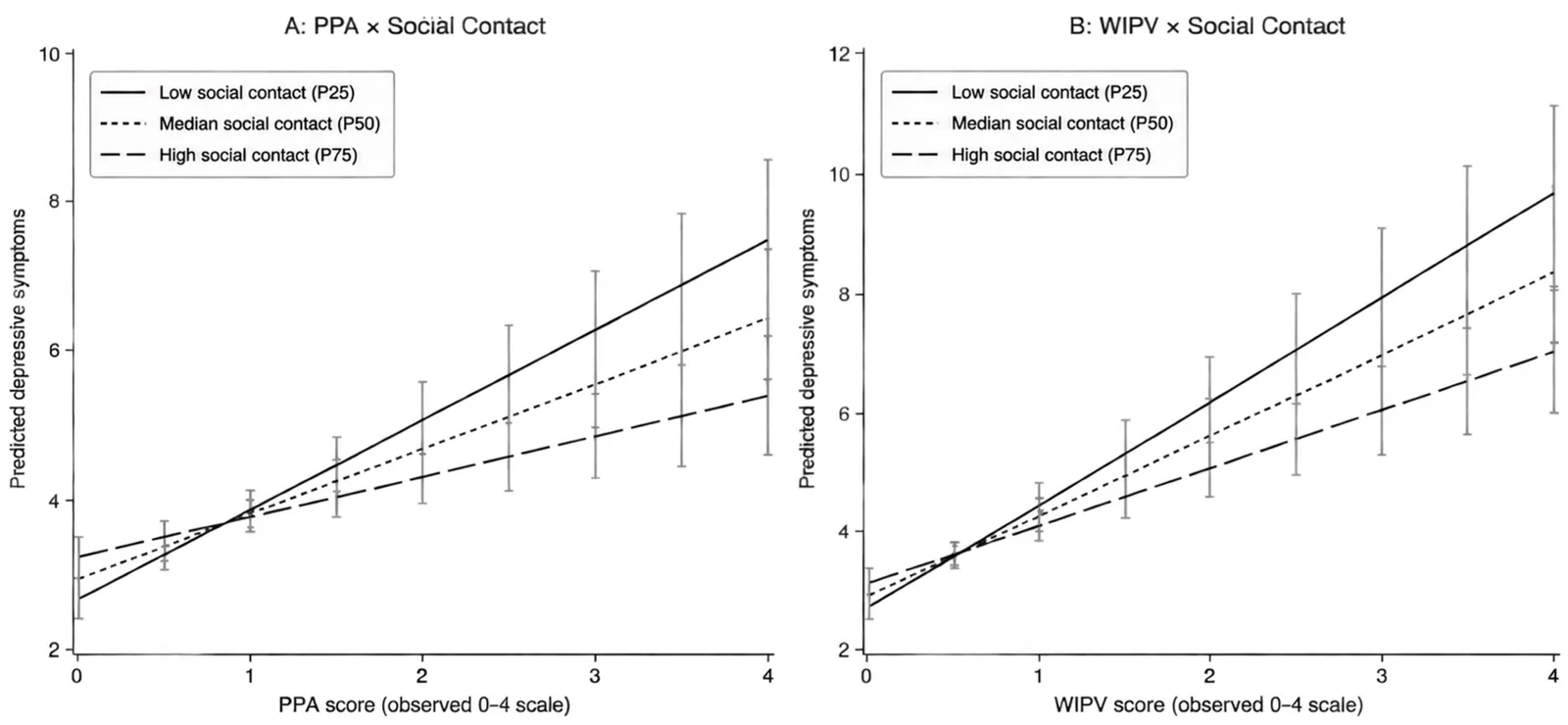
Moderating Effects of Social Contact Frequency on the CFV–Depression Association. Note: Predicted values were estimated from OLS moderation models with control variables held constant. The x-axes show the observed 0–4 scales of PPA and WIPV. Solid, short-dashed, and long-dashed lines denote low (P25), median (P50), and high (P75) social contact frequency, respectively. Vertical caps indicate 95% confidence intervals. CFV = childhood family violence; PPA = parental physical abuse; WIPV = witnessing interparental violence.

Figure 1 illustrates the interaction patterns using the observed 0–4 ranges of PPA and WIPV rather than extrapolated standardized values. Predicted values were estimated at the 25th, 50th, and 75th percentiles of social contact frequency and include 95% confidence intervals. Simple-slope analyses showed that the association between PPA and depressive symptoms was strongest at low social contact frequency (b = 1.101, 95% CI [0.820, 1.382]), weaker at median contact (b = 0.812, 95% CI [0.583, 1.040]), and weakest at high contact (b = 0.496, 95% CI [0.283, 0.710]). A similar pattern was observed for WIPV: the slope was strongest at low contact (b = 1.516, 95% CI [1.154, 1.877]), weaker at median contact (b = 1.197, 95% CI [0.905, 1.489]), and weakest at high contact (b = 0.850, 95% CI [0.602, 1.097]). These patterns reflect differences in depressive symptom scores on the PHQ-9 scale and should not be interpreted as differences in clinical diagnosis. In practical terms, the difference between the low- and high-contact simple slopes was 0.605 PHQ-9 points for PPA and 0.666 points for WIPV, indicating that the magnitude of the moderation was modest. Johnson–Neyman-style plots restricted to the observed range of social contact frequency are provided in Supplementary Figures S1 and S2.

### 4.4. Unique Associations of WIPV and PPA

Table 5 presents the combined models examining the unique associations of WIPV and PPA with depressive symptoms. As shown in Table 2, WIPV and PPA were highly correlated (r = 0.762, *p* < 0.001), indicating considerable shared variance between the two exposures. Multicollinearity diagnostics showed the largest VIF was 2.87 and the mean VIF was 1.52, below conventional concern thresholds; however, the coefficient correlation between WIPV and PPA in the combined model was substantial (r = −0.710). Thus, comparisons between WIPV and PPA should be interpreted cautiously as model-specific adjusted associations rather than definitive evidence that one form is intrinsically more harmful than the other.

In Model 6, WIPV remained significantly and positively associated with depressive symptoms (b = 0.704, *p* < 0.001), whereas the association between PPA and depressive symptoms was attenuated and no longer statistically significant (b = 0.120, *p* > 0.05). Given the substantial overlap between PPA and WIPV, this pattern should be interpreted as a model-specific unique association rather than evidence that PPA is unimportant or that WIPV is intrinsically more harmful. A supplementary joint-exposure analysis (Table S9, Model 3) showed that respondents reporting both PPA and WIPV had the highest predicted depressive symptoms among all exposure groups, underscoring the importance of co-occurrence.

Models 7 and 8 added the interaction terms separately—Model 7 added PPA × social contact frequency while controlling for WIPV, and Model 8 added WIPV × social contact frequency while controlling for PPA. This specification avoids estimating two interaction terms from highly correlated measures in the same equation. In Model 7, PPA × social contact frequency was negative and significant (b = −0.533, SE = 0.083, *p* < 0.001). In Model 8, WIPV × social contact frequency was also negative and significant (b = −0.487, SE = 0.082, *p* < 0.001). Thus, each interaction remained significant when the other violence exposure’s main effect was controlled. Because the two interactions were not estimated simultaneously, however, these models do not establish independent moderation effects.

### 4.5. Robustness and Sensitivity Analyses

Robustness checks are summarized in Supplementary Tables S6–S14 and Figures S3–S7. The negative interactions remained statistically significant with maximum-exposure scoring, after omitting self-rated health, after excluding influential observations, and across alternative PHQ-9 specifications; Breusch–Pagan tests were significant in every primary model (all *p* < 0.001), supporting the use of HC1 robust standard errors, and the maximum Cook’s distances were 0.055 and 0.052 in the PPA and WIPV interaction models, respectively. Domain-specific models yielded similar negative interactions for family contact (PPA b = −0.432; WIPV b = −0.475), friend contact (PPA b = −0.421; WIPV b = −0.481), and social participation (PPA b = −0.294; WIPV b = −0.369; all *p* < 0.001). Family-contact main associations were not statistically significant, whereas friend contact and social participation were negatively associated with PHQ-9 scores in the domain-specific models. Requiring at least 1, 3, 5, or all 7 valid contact items produced negative interaction estimates for PPA (−0.442, −0.411, −0.439, and −0.547) and WIPV (−0.487, −0.465, −0.461, and −0.527), and the full observed 0–4 range of both violence measures appeared in every contact quintile.

Additional adjustment for paternal and maternal education at age 12 yielded substantively unchanged results in the reduced sample (N = 1732): the PPA × social contact frequency interaction remained negative and significant (b = −0.499, *p* < 0.001), as did the WIPV × social contact frequency interaction (b = −0.475, *p* < 0.001). Separate-city estimates were negative in both settings; the PPA interaction was stronger in Beijing (b = −0.686) than in Chengdu (b = −0.214; three-way *p* < 0.001), whereas the WIPV difference was not statistically significant (b = −0.697 vs. −0.451; *p* = 0.111).

## 5. Discussion

Drawing on a sample of ever-married women in Beijing and Chengdu, this study examined associations between childhood family violence and depressive symptoms and the moderating role of social contact frequency. Consistent with Hypotheses 1a and 1b, both PPA and WIPV were positively associated with adult depressive symptoms in separate models. These findings are consistent with a large body of research on adverse childhood experiences documenting enduring associations between childhood exposure to family violence and mental health problems later in life (Felitti et al., 1998; Mandelli et al., 2015), as well as evidence from Chinese populations (Y. Li & Lu, 2020; Du et al., 2022; Lv & Li, 2023). From a life-course perspective, the present findings underscore the enduring relevance of early family adversity for psychological well-being in adulthood. Although childhood family violence necessarily preceded the assessment of adult depressive symptoms, the retrospective and cross-sectional design does not permit causal inference regarding the developmental processes linking these experiences over time. The separate PPA and WIPV models are therefore interpreted as total association models because PPA and WIPV were not entered simultaneously until the combined-model analyses.

Contrary to Hypothesis 2, social contact frequency was not independently associated with depressive symptoms after accounting for childhood family violence and covariates. However, significant negative interaction effects emerged for both PPA and WIPV in the separate moderation models, supporting Hypotheses 3a and 3b and a pattern consistent with the stress-buffering hypothesis. According to this perspective, social resources may be particularly relevant under conditions of heightened stress, rather than conferring uniform benefits across all individuals (Cohen & Wills, 1985; Pearlin, 2010). Consistent with this framework, higher social contact frequency attenuated the positive associations of both PPA and WIPV with depressive symptoms, with the associations between childhood family violence and depressive symptoms becoming progressively weaker at higher levels of social contact frequency. Thus, the absence of an overall main association for social contact frequency does not contradict its moderating role; rather, the association between social contact frequency and depressive symptoms appears to vary according to the level of childhood family violence exposure. At the same time, the positive bivariate correlations between social contact frequency and both violence measures caution against viewing frequent contact as uniformly beneficial and may reflect family structure, compensatory social engagement, urban social patterns, or features of the contact-frequency measure. Because the present data are cross-sectional, the observed interactions should be interpreted as statistical moderation rather than evidence that greater social contact causally reduces depressive symptoms associated with childhood family violence.

The combined models represent a key contribution of this study by helping to disentangle the unique associations of the two violence forms. In these models, WIPV retained a significant unique association with depressive symptoms after adjustment for PPA, whereas the PPA coefficient was attenuated. This pattern may suggest that WIPV captures broader aspects of family conflict and emotional insecurity that remain relevant net of direct physical victimization. However, this finding should be viewed as tentative and model-specific: PPA and WIPV were highly correlated and frequently co-occurred, and the attenuation of the PPA coefficient may reflect shared variance rather than substantive unimportance. Indeed, the joint-exposure analysis showed that co-exposure to both forms was associated with the highest depressive symptoms, underscoring the need to consider overlap rather than treating them as entirely separable experiences. In the split interaction models, both moderation terms remained significant when entered separately, suggesting that the negative interaction pattern persisted for each form of violence after adjusting for the other’s main effect. Taken together, the combined-model findings refine rather than replace the separate-model tests, and should be treated as model-specific associations requiring replication.

Finally, sensitivity analyses indicated that the PPA moderation pattern was stronger in Beijing than in Chengdu, whereas no significant city difference was observed for WIPV. Although this heterogeneity suggests that the magnitude of the PPA interaction may vary across urban contexts, it does not undermine the overall moderation findings but qualifies their consistency across settings.

### 5.1. Implications of the Study

The findings offer several directions for future research and intervention development. First, the observed moderation pattern suggests that safe and supportive social ties warrant further investigation as a potential source of variation in the association between childhood family violence and adult depressive symptoms. Future longitudinal and intervention research could examine whether changes in social contact frequency or social engagement are associated with subsequent changes in depressive symptoms among women with histories of childhood family violence. However, the results do not imply that simply increasing contact will improve mental health, particularly because family contact may be ambivalent, stressful, or even unsafe for individuals with histories of family violence. Future intervention research should therefore assess relationship quality, perceived safety, conflict, and the availability of emotional and instrumental support when evaluating whether and how changes in social contact might influence mental health.

Second, the stronger unique association of WIPV in the combined models, alongside the joint-exposure finding that co-occurring violence was linked to the highest depressive symptoms, underscores the importance of family-level prevention efforts that reduce children’s exposure to both direct parental physical abuse and interparental violence. Programs promoting healthy conflict resolution and fostering a stable, nonviolent family environment may be particularly critical. Given that the present study did not evaluate any intervention, these implications should be understood as hypotheses for future program development and rigorous testing rather than as evidence of intervention effectiveness.

### 5.2. Limitations

Several limitations should be acknowledged. First, the cross-sectional design and reliance on retrospective self-reports of childhood experiences limit the ability to draw causal conclusions. Although reverse causation is unlikely given the temporal ordering of childhood violence and adult depressive symptoms, the possibility of mood-congruent recall bias cannot be ruled out, as individuals experiencing depressive symptoms may recall childhood experiences more negatively. Future research should employ longitudinal and prospective study designs to more rigorously establish temporal ordering and better understand the mechanisms linking childhood family violence to mental health outcomes in adulthood.

Second, the violence measures were limited to brief retrospective items that may capture a range of experiences from culturally normalized corporal punishment to more severe physical abuse, and the strong correlation between PPA and WIPV made it difficult to fully disentangle their unique associations. Future research should use more detailed measures of severity, chronicity, perpetrator identity, and developmental timing to better distinguish these related but distinct forms of exposure.

Third, the measure of social contact frequency was limited to the frequency of in-person interactions and social gatherings, which did not capture qualitative dimensions of social relationships, such as perceived support, relationship quality, safety, or the availability of emotional and instrumental resources. Future research should distinguish structural social contact from functional social support and separately examine family, friend, and community domains, particularly because family contact may not be uniformly beneficial for individuals exposed to family violence.

Fourth, the sample was restricted to ever-married women residing in Beijing and Chengdu, which limits generalizability to unmarried women, men, rural residents, and populations in other regions of China. Sampling weights and design variables were not available. In addition, listwise deletion may have introduced selection bias, given that included and excluded respondents differed on several observed characteristics, including age, education, household income, depressive symptoms, childhood family violence, social contact frequency, and city of residence (Supplementary Table S1).

Fifth, residual confounding by other childhood and adult circumstances remains possible. Although the primary models adjusted for current education and household income, and sensitivity analyses additionally adjusted for parental education at age 12, other adverse childhood experiences and adult intimate partner violence were not included as routine covariates because they may reflect overlapping childhood adversity or later-life pathways rather than straightforward baseline confounders (Chan et al., 2018; Xu et al., 2019). Accordingly, the reported associations should not be interpreted as fully isolated from all co-occurring childhood adversities or later-life experiences.

Finally, although robust standard errors and alternative specifications produced similar patterns, all study measures relied on self-reports, raising the possibility of shared method bias; PHQ-9 is a screening scale rather than a diagnostic interview.

## 6. Conclusions

This study examined the associations between childhood family violence and depressive symptoms in adulthood and the moderating role of social contact frequency among ever-married women in Beijing and Chengdu, China. The findings indicate that both parental physical abuse and witnessing interparental violence are associated with elevated levels of depressive symptoms in adulthood in separate models, although their unique associations were difficult to fully separate given their substantial overlap. Moreover, higher social contact frequency attenuated the positive associations between childhood family violence and depressive symptoms, a pattern consistent with the stress-buffering hypothesis rather than a uniformly beneficial association across individuals. However, given the cross-sectional and retrospective design, these findings should be interpreted as conditional associations rather than evidence of causal protection. Collectively, these findings highlight the enduring associations between childhood family violence and adult mental health and underscore the potential importance of social contact in understanding variation in psychological well-being across the life course.

## Figures and Tables

**Table 1:** Descriptive Statistics for All Focal and Control Variables (*N* = 1849).

Variable	M	SD	Min	Max
Dependent Variable				
Depressive symptoms (PHQ-9 total)	3.52	3.76	0	22
Independent Variables				
Witnessing interparental violence (WIPV)	0.48	0.87	0	4
Parental physical abuse (PPA)	0.70	0.90	0	4
Moderator				
Social contact frequency	1.92	0.80	0	4
Control Variables				
Age (years)	47.05	13.52	21	75
Years of education	11.89	3.93	0	18
Log household income	8.995	0.859	5.298	11.918
Married (1 = yes)	0.879	0.326	0	1
Self-rated health (1–7)	5.16	0.94	1	7
Urban hukou (1 = yes)	0.693	0.461	0	1
Ethnic minority (1 = yes)	0.023	0.149	0	1
CCP member (1 = yes)	0.107	0.309	0	1

Note: PHQ-9 = 9-item Patient Health Questionnaire; WIPV = Witnessing interparental violence; PPA = Parental physical abuse; CCP = Chinese Communist Party.

**Table 2:** Correlations among Focal Variables (*N* = 1849).

Variables	1	2	3	4
1. Depressive Symptoms (PHQ-9)	1			
2. Witnessing interparental violence (WIPV)	0.300 ***	1		
3. Parental physical abuse (PPA)	0.248 ***	0.762 ***	1	
4. Social contact frequency	−0.046 *	0.153 ***	0.128 ***	1

Note: * *p* < 0.05, *** *p* < 0.001.

**Table 3:** Main Effects of CFV and Social Contact Frequency on Depressive Symptoms (*N* = 1849).

Variable	Model 1 (Baseline)	Model 2 (PPA)	Model 3 (WIPV)
PPA		0.585 *** [0.363, 0.808]	
WIPV			0.795 *** [0.539, 1.051]
Social contact frequency	0.009 [−0.182, 0.200]	−0.091 [−0.284, 0.102]	−0.151 [−0.346, 0.043]
Age (centered)	0.003 [−0.014, 0.020]	0.004 [−0.013, 0.021]	0.002 [−0.015, 0.019]
Urban hukou	−1.230 *** [−1.658, −0.802]	−0.937 *** [−1.357, −0.517]	−0.767 *** [−1.192, −0.342]
Ethnic minority	4.784 *** [3.062, 6.507]	4.457 *** [2.763, 6.150]	4.209 *** [2.562, 5.855]
CCP member	1.803 *** [1.117, 2.489]	1.467 *** [0.761, 2.174]	1.248 *** [0.548, 1.947]
Education (centered)	−0.086 ** [−0.149, −0.023]	−0.068 * [−0.130, −0.006]	−0.062 [−0.124, 0.001]
Married	−0.549 * [−1.090, −0.008]	−0.497 [−1.024, 0.031]	−0.496 [−1.013, 0.021]
Self-rated health	−0.542 *** [−0.762, −0.322]	−0.499 *** [−0.716, −0.281]	−0.531 *** [−0.749, −0.313]
Log household income (centered)	−0.006 [−0.286, 0.274]	0.051 [−0.220, 0.322]	0.050 [−0.224, 0.325]
Constant	7.438 *** [6.174, 8.702]	6.983 *** [5.736, 8.230]	6.953 *** [5.704, 8.202]
R^2^	0.135	0.154	0.166

Note: Cells report unstandardized OLS coefficients with 95% confidence intervals in brackets. Confidence intervals and significance tests are based on heteroskedasticity-robust standard errors. PPA, WIPV, and social contact frequency were standardized. All models adjust for age, urban hukou, ethnic minority status, CCP membership, education, household income, marital status, self-rated health, and city. * *p* < 0.05, ** *p* < 0.01, *** *p* < 0.001.

**Table 4:** Interaction Effects of Social Contact Frequency and CFV on Depressive Symptoms (*N* = 1849).

Variable	Model 4 (PPA)	Model 5 (WIPV)
PPA	0.776 *** [0.551, 1.002]	
WIPV		1.157 *** [0.872, 1.443]
Social contact frequency	0.072 [−0.108, 0.252]	0.029 [−0.149, 0.207]
PPA × Social contact frequency	−0.442 *** [−0.595, −0.289]	
WIPV × Social contact frequency		−0.487 *** [−0.648, −0.326]
Controls	Included	Included
R^2^	0.177	0.195

Note: Cells report unstandardized OLS coefficients with 95% confidence intervals in brackets. Confidence intervals and significance tests are based on heteroskedasticity-robust standard errors. Controls include age, urban hukou, ethnic minority status, CCP membership, education, household income, marital status, self-rated health, and city. PPA, WIPV, and social contact frequency were standardized. *** *p* < 0.001.

**Table 5:** Unique Associations of WIPV and PPA with Depressive Symptoms (*N* = 1849).

Variable	Model 6 (Main Effects)	Model 7 (PPA Interaction)	Model 8 (WIPV Interaction)
Social contact frequency	−0.153 [−0.348, 0.041]	0.022 [−0.160, 0.203]	0.027 [−0.151, 0.205]
PPA	0.120 [−0.201, 0.441]	0.189 [−0.132, 0.510]	0.122 [−0.186, 0.430]
WIPV	0.704 *** [0.342, 1.066]	0.947 *** [0.568, 1.327]	1.064 *** [0.685, 1.444]
PPA × Social contact frequency		−0.533 *** [−0.696, −0.369]	
WIPV × Social contact frequency			−0.487 *** [−0.649, −0.325]
Controls	Included	Included	Included
R^2^	0.166	0.198	0.196

Note: Cells report unstandardized OLS coefficients with 95% confidence intervals in brackets. Confidence intervals and significance tests are based on heteroskedasticity-robust standard errors. Models 7 and 8 estimate the PPA and WIPV interactions separately; no model includes both interaction terms simultaneously. Controls include age, urban hukou, ethnic minority status, CCP membership, education, household income, marital status, self-rated health, and city. *** *p* < 0.001.

## Data Availability

Due to confidentiality agreements with participants, the dataset is not publicly available. However, access may be granted upon reasonable request to the corresponding author.

## Supplementary Materials

The following supporting information can be downloaded at: https://www.mdpi.com/article/10.3390/bs16091698/s1.
