# Supplementary material for: Childhood Family Violence and Depressive Symptoms in Urban Chinese Women: The Moderating Role of Social Contact Frequency
Source: Behav Sci (Basel). 2026 Sep 20;16(9):1698. doi: 10.3390/bs16091698 (PMC13603159; doi:10.3390/bs16091698)
Supplement: Supplementary file 1 [file behavsci-16-01698-s001.zip › behavsci-4482847-supplementary.pdf]

Supplementary Materials

Manuscript: Childhood Family Violence and Depressive Symptoms among Ever-Married Women in Two Urban Chinese Settings: The Moderating Role of Social Contact Frequency

These supplementary tables and figures correspond to additional analyses conducted in response to reviewer comments on missing data, measurement, model diagnostics, interaction probing, multicollinearity, and robustness checks. Updated analyses adjust for respondent-and-spouse monthly income; Table S12 additionally adjusts for paternal and maternal education when the respondent was age 12.

Table S1a. Sample flow

Step	N	Percent of original sample	Description
Original survey sample	2,086	100.00%	Ever-married women interviewed in the 2020 Family and Health Survey
Excluded because age was missing or >75	18	0.86%	Age restriction used in the analytic sample
Retained after age restriction	2,068	99.14%	Cases eligible after age restriction
Excluded because of missing core analysis variables	219	10.50%	Missing focal variables or covariates
Final analytic sample	1,849	88.64%	Cases included in the main regression analyses

Table S1b. Variable-specific missingness before final analytic sample

Variable	Missing N	Nonmissing N	Missing %	Observed range
WIPV mean score	183	1,903	8.77%	0-4
PPA mean score	148	1,938	7.09%	0-4
Social contact frequency	48	2,038	2.30%	0-4
Hukou status	1	2,085	0.05%	0-1
Other focal variables and covariates	0	2,086	0.00%	--

Table S1c. Included versus excluded cases before listwise deletion

Variable	Excluded	Included	Test / p-value
Age, mean (SD)	51.38 (16.07), n=237	47.05 (13.52), n=1,849	t=4.54, p<.001
Years of education, mean (SD)	11.18 (4.37), n=237	11.89 (3.93), n=1,849	t=-2.59, p=.010
Family income, yuan/month, mean (SD)	10,537.75 (11,075.29), n=237	10,956.53 (10,152.47), n=1,849	t=-0.59, p=.554
PHQ-9, mean (SD)	2.97 (4.11), n=237	3.52 (3.76), n=1,849	t=-2.12, p=.034
WIPV mean score, mean (SD)	1.66 (1.63), n=54	0.49 (0.87), n=1,849	t=9.38, p<.001
PPA mean score, mean (SD)	1.05 (1.31), n=89	0.70 (0.92), n=1,849	t=3.40, p=.001
Social contact frequency, mean (SD)	1.73 (0.88), n=189	1.92 (0.80), n=1,849	t=-3.17, p=.002
Beijing, n (%)	190 (80.17%)	1,075 (58.14%)	chi-square=42.71, p<.001
Chengdu, n (%)	47 (19.83%)	774 (41.86%)	chi-square=42.71, p<.001
Ethnic minority, n (%)	5 (2.11%)	42 (2.27%)	chi-square=0.03, p=.874
CCP member, n (%)	29 (12.24%)	197 (10.65%)	chi-square=0.54, p=.461

Table S1d. Number of valid social contact items

Number of valid items	N	Percent	Cumulative percent
0	48	2.30	2.30
1	16	0.77	3.07
2	16	0.77	3.84

3	22	1.05	4.89
4	15	0.72	5.61
5	508	24.35	29.96
6	55	2.64	32.60
7	1,406	67.40	100.00

Table S1e. "Not applicable" responses in social contact items

Item	Question domain	Not applicable N	Not applicable %
Q186	Respondent visits parents	414	19.85
Q187	Respondent visits siblings	279	13.37
Q188	Respondent visits friends	96	4.60
Q189	Parents visit respondent	439	21.05
Q190	Siblings visit respondent	278	13.33
Q191	Friends visit respondent	100	4.79
Q192	Formal or informal gatherings outside family	110	5.27

Table S2. Item distributions for childhood violence variables

Item	Response category	N	Percent
Q194 Father hit mother	Very frequently	77	3.69
Q194 Father hit mother	Frequently	51	2.44
Q194 Father hit mother	Sometimes	160	7.67
Q194 Father hit mother	Rarely	349	16.73
Q194 Father hit mother	Never	1239	59.40
Q194 Father hit mother	Not applicable	210	10.07
Q195 Mother hit father	Very frequently	62	2.97
Q195 Mother hit father	Frequently	39	1.87
Q195 Mother hit father	Sometimes	84	4.03
Q195 Mother hit father	Rarely	290	13.90
Q195 Mother hit father	Never	1418	67.98
Q195 Mother hit father	Not applicable	193	9.25
Q196 Hit by father	Very frequently	60	2.88
Q196 Hit by father	Frequently	54	2.59
Q196 Hit by father	Sometimes	197	9.44
Q196 Hit by father	Rarely	485	23.25
Q196 Hit by father	Never	1107	53.07
Q196 Hit by father	Not applicable	183	8.77
Q197 Hit by mother	Very frequently	55	2.64
Q197 Hit by mother	Frequently	85	4.07
Q197 Hit by mother	Sometimes	227	10.88
Q197 Hit by mother	Rarely	528	25.31
Q197 Hit by mother	Never	1024	49.09
Q197 Hit by mother	Not applicable	167	8.01

Table S3. Correlation matrix from supplementary analyses

	(1)												
	**PHQ-9 depressive symptoms total score, complete 9 items, 0-27**	**WIPV mean score, standardized**	**PPA mean score, standardized**	**Social contact frequency, standardized**	**Age centered**	**Urban/non-agricultural hukou**	**Ethnic minority**	**CCP member**	**Education centered**	**Currently married or remarried**	**Self-rated health, reversed 1-7**	**Log monthly income centered**	**City: 1 Chengdu, 0 Beijing**
PHQ-9 depressive symptoms total score, complete 9 items, 0-27	1												
WIPV mean score, standardized	0.304***	1											
PPA mean score, standardized	0.255***	0.763***	1										
Social contact frequency, standardized	-0.0461*	0.152***	0.131***	1									
Age centered	0.126***	0.0807***	0.0598*	-0.197***	1								
Urban/non-agricultural hukou	-0.209***	-0.359***	-0.301***	0.122***	-0.00739	1							
Ethnic minority	0.191***	0.133***	0.0909***	-0.0158	0.0298	-0.00882	1						
CCP member	0.149***	0.305***	0.230***	0.195***	-0.000391	-0.101***	-0.0174	1					
Education centered	-0.187***	-0.229***	-0.218***	0.119***	-0.425***	0.344***	-0.0208	-0.00477	1				
Currently married or remarried	-0.0864***	-0.0771***	-0.0747**	0.0697**	-0.204***	0.0550*	-0.0101	0.0476*	0.159***	1			
Self-rated health, reversed 1-7	-0.150***	0.00504	-0.0436	0.270***	-0.437***	0.0240	0.0206	0.0949***	0.212***	0.121***	1		
Log household income centered	-0.134***	-0.179***	-0.183***	0.119***	-0.388***	0.244***	-0.0618**	0.0151	0.414***	0.323***	0.218***	1	
City: 1 Chengdu, 0 Beijing	-0.103***	-0.260***	-0.105***	0.143***	-0.0561*	0.184***	-0.122***	-0.147***	-0.00873	0.103***	0.0116	0.0343	1

Note: Pearson correlations are reported. * $p < .05$, ** $p < .01$, *** $p < .001$.

Table S4. Main effects with heteroskedasticity-robust standard errors

	(1)	(2)	(3)
	PHQ-9 depressive symptoms total score, complete 9 items, 0-27	PHQ-9 depressive symptoms total score, complete 9 items, 0-27	PHQ-9 depressive symptoms total score, complete 9 items, 0-27
Social contact frequency, standardized	0.009	-0.091	-0.151
	(0.097)	(0.098)	(0.099)
Age centered	0.003	0.004	0.002
	(0.009)	(0.009)	(0.009)
Urban/non-agricultural hukou	-1.230***	-0.937***	-0.767***
	(0.218)	(0.214)	(0.217)
Ethnic minority	4.784***	4.457***	4.209***
	(0.878)	(0.863)	(0.839)
CCP member	1.803***	1.467***	1.248***
	(0.350)	(0.360)	(0.357)
Education centered	-0.086**	-0.068*	-0.062
	(0.032)	(0.032)	(0.032)
Currently married or remarried	-0.549*	-0.497	-0.496
	(0.276)	(0.269)	(0.264)
Self-rated health, reversed 1-7	-0.542***	-0.499***	-0.531***
	(0.112)	(0.111)	(0.111)
Log household income centered	-0.006	0.051	0.050
	(0.143)	(0.138)	(0.140)
City: 1 Chengdu, 0 Beijing	-0.183	-0.128	0.122
	(0.161)	(0.158)	(0.159)
PPA mean score, standardized		0.585***	
		(0.113)	
WIPV mean score, standardized			0.795***
			(0.131)
Constant	7.438***	6.983***	6.953***
	(0.645)	(0.636)	(0.637)
Observations	1849	1849	1849
R2	0.135	0.154	0.166
Adjusted R2	0.130	0.149	0.161

Note: Unstandardized coefficients are reported with standard errors in parentheses unless otherwise indicated. * p < .05, ** p < .01, *** p < .001.

Table S5. Interaction models with heteroskedasticity-robust standard errors

	(1)	(2)
	**PHQ-9 depressive symptoms total score, complete 9 items, 0-27**	**PHQ-9 depressive symptoms total score, complete 9 items, 0-27**
PPA mean score, standardized	0.776***	
	(0.115)	
Social contact frequency, standardized	0.072	0.029
	(0.092)	(0.091)
PPA mean score, standardized # Social contact frequency, standardized	-0.442***	
	(0.078)	
Age centered	0.007	0.005
	(0.008)	(0.009)
Urban/non-agricultural hukou	-0.911***	-0.710***
	(0.214)	(0.215)
Ethnic minority	4.583***	4.222***
	(0.844)	(0.805)
CCP member	1.731***	1.500***
	(0.360)	(0.351)
Education centered	-0.067*	-0.054
	(0.030)	(0.030)
Currently married or remarried	-0.409	-0.372
	(0.278)	(0.275)
Self-rated health, reversed 1-7	-0.427***	-0.482***
	(0.110)	(0.111)
Log household income centered	0.036	0.026
	(0.137)	(0.140)
City: 1 Chengdu, 0 Beijing	-0.227	0.079
	(0.160)	(0.160)
WIPV mean score, standardized		1.157***
		(0.146)
WIPV mean score, standardized # Social contact frequency, standardized		-0.487***
		(0.082)
Constant	6.590***	6.620***
	(0.636)	(0.645)
Observations	1849	1849
R2	0.177	0.195
Adjusted R2	0.172	0.190

Note: Unstandardized coefficients are reported with standard errors in parentheses unless otherwise indicated. * p < .05, ** p < .01, *** p < .001.

Table S6. Sensitivity analysis using maximum exposure scores

	(1)	(2)	(3)	(4)
	**PHQ-9 depressive symptoms total score, complete 9 items, 0-27**	**PHQ-9 depressive symptoms total score, complete 9 items, 0-27**	**PHQ-9 depressive symptoms total score, complete 9 items, 0-27**	**PHQ-9 depressive symptoms total score, complete 9 items, 0-27**
PPA maximum exposure, standardized	0.666^{***}		0.809^{***}	
	(0.107)		(0.109)	
Social contact frequency, standardized	-0.087	-0.128	0.055	0.030
	(0.098)	(0.099)	(0.092)	(0.089)
Age centered	0.004	0.001	0.006	0.003
	(0.009)	(0.009)	(0.008)	(0.008)
Urban/non-agricultural hukou	-0.922^{***}	-0.697^{**}	-0.894^{***}	-0.680^{**}
	(0.212)	(0.215)	(0.212)	(0.213)
Ethnic minority	4.409^{***}	4.099^{***}	4.530^{***}	4.140^{***}
	(0.849)	(0.821)	(0.833)	(0.800)
CCP member	1.450^{***}	1.241^{***}	1.689^{***}	1.480^{***}
	(0.354)	(0.352)	(0.354)	(0.350)
Education centered	-0.063^{*}	-0.052	-0.059	-0.042
	(0.032)	(0.032)	(0.031)	(0.030)
Currently married or remarried	-0.470	-0.447	-0.373	-0.332
	(0.270)	(0.263)	(0.280)	(0.275)
Self-rated health, reversed 1-7	-0.521^{***}	-0.555^{***}	-0.477^{***}	-0.529^{***}
	(0.111)	(0.110)	(0.110)	(0.110)
Log household income centered	0.068	0.062	0.057	0.047
	(0.138)	(0.139)	(0.138)	(0.140)
City: 1 Chengdu, 0 Beijing	-0.176	0.151	-0.261	0.108
	(0.159)	(0.159)	(0.160)	(0.159)
WIPV maximum exposure, standardized		0.926^{***}		1.121^{***}
		(0.124)		(0.126)
PPA maximum exposure, standardized # Social contact frequency, standardized			-0.419^{***}	
			(0.080)	
WIPV maximum exposure, standardized # Social contact frequency, standardized				-0.408^{***}
				(0.080)
Constant	7.089^{***}	6.979^{***}	6.810^{***}	6.762^{***}
	(0.632)	(0.631)	(0.632)	(0.634)
Observations	1849	1849	1849	1849
R2	0.161	0.178	0.179	0.198
Adjusted R2	0.156	0.174	0.174	0.193

Note: Unstandardized coefficients are reported with standard errors in parentheses unless otherwise indicated. * p < .05, ** p < .01, *** p < .001.

Table S7. Supplementary models by social contact domains

	(1)	(2)	(3)	(4)	(5)	(6)
	PHQ-9 depressive symptoms total score, complete 9 items, 0-27	PHQ-9 depressive symptoms total score, complete 9 items, 0-27	PHQ-9 depressive symptoms total score, complete 9 items, 0-27	PHQ-9 depressive symptoms total score, complete 9 items, 0-27	PHQ-9 depressive symptoms total score, complete 9 items, 0-27	PHQ-9 depressive symptoms total score, complete 9 items, 0-27
PPA mean score, standardized	0.725***	0.781***	0.723***			
	(0.118)	(0.112)	(0.113)			
Standardized values of family_contact	0.147			0.106		
	(0.090)			(0.089)		
PPA mean score, standardized # Standardized values of family_contact	-0.432***					
	(0.093)					
Age centered	0.009	0.012	0.011	0.007	0.009	0.008
	(0.009)	(0.008)	(0.008)	(0.009)	(0.008)	(0.008)
Urban/non-agricultural hukou	-0.942***	-0.838***	-0.836***	-0.768***	-0.631**	-0.618**
	(0.217)	(0.212)	(0.211)	(0.218)	(0.212)	(0.214)
Ethnic minority	4.564***	4.473***	4.668***	4.247***	4.050***	4.243***
	(0.849)	(0.900)	(0.940)	(0.820)	(0.846)	(0.899)
CCP member	1.638***	1.657***	1.599***	1.413***	1.365***	1.322***
	(0.367)	(0.348)	(0.353)	(0.362)	(0.332)	(0.339)
Education centered	-0.064*	-0.062*	-0.052	-0.052	-0.048	-0.036
	(0.031)	(0.029)	(0.030)	(0.031)	(0.029)	(0.029)
Currently married or remarried	-0.438	-0.451	-0.594*	-0.425	-0.418	-0.539
	(0.277)	(0.282)	(0.281)	(0.273)	(0.276)	(0.278)
Self-rated health, reversed 1-7	-0.465***	-0.300**	-0.341**	-0.514***	-0.360***	-0.389***
	(0.111)	(0.109)	(0.106)	(0.112)	(0.109)	(0.105)
Log household income centered	0.028	0.079	0.115	0.032	0.072	0.099
	(0.139)	(0.136)	(0.137)	(0.141)	(0.139)	(0.139)
City: 1 Chengdu, 0 Beijing	-0.217	-0.075	-0.087	0.066	0.226	0.220
	(0.160)	(0.157)	(0.153)	(0.160)	(0.158)	(0.152)
Standardized values of friend_contact		-0.187*			-0.211*	
		(0.087)			(0.084)	
PPA mean score, standardized # Standardized values of friend_contact		-0.421***				
		(0.073)				
Standardized values of social_participation			-0.287***			-0.314***
			(0.082)			(0.080)
PPA mean score, standardized # Standardized values of social_participation			-0.294***			
			(0.070)			
WIPV mean score, standardized				1.068***	1.163***	1.097***
				(0.148)	(0.137)	(0.136)
WIPV mean score, standardized # Standardized values of family_contact				-0.475***		
				(0.102)		
WIPV mean score, standardized # Standardized values of friend_contact					-0.481***	
					(0.075)	
WIPV mean score, standardized # Standardized values of						-0.367***

	(1)	(2)	(3)	(4)	(5)	(6)
	**PHQ-9 depressive symptoms total score, complete 9 items, 0-27**	**PHQ-9 depressive symptoms total score, complete 9 items, 0-27**	**PHQ-9 depressive symptoms total score, complete 9 items, 0-27**	**PHQ-9 depressive symptoms total score, complete 9 items, 0-27**	**PHQ-9 depressive symptoms total score, complete 9 items, 0-27**	**PHQ-9 depressive symptoms total score, complete 9 items, 0-27**
social_participation						
						(0.070)
Constant	6.837***	5.816***	6.134***	6.877***	5.879***	6.102***
	(0.635)	(0.637)	(0.613)	(0.645)	(0.635)	(0.611)
Observations	1833	1828	1805	1833	1828	1805
R2	0.170	0.177	0.161	0.184	0.201	0.185
Adjusted R2	0.164	0.172	0.155	0.179	0.196	0.179

Note: Unstandardized coefficients are reported with standard errors in parentheses unless otherwise indicated. * $p < .05$, ** $p < .01$, *** $p < .001$.

Table S8. Interaction models excluding influential observations

	(1)	(2)
	PHQ-9 depressive symptoms total score, complete 9 items, 0-27	PHQ-9 depressive symptoms total score, complete 9 items, 0-27
PPA mean score, standardized	0.532***	
	(0.089)	
Social contact frequency, standardized	0.123	0.074
	(0.075)	(0.074)
PPA mean score, standardized # Social contact frequency, standardized	-0.269***	
	(0.059)	
Age centered	0.012	0.007
	(0.006)	(0.006)
Urban/non-agricultural hukou	-0.649***	-0.473**
	(0.175)	(0.177)
Ethnic minority	4.742***	4.494***
	(0.474)	(0.433)
CCP member	1.307***	1.144***
	(0.272)	(0.273)
Education centered	-0.031	-0.016
	(0.023)	(0.023)
Currently married or remarried	-0.239	-0.146
	(0.215)	(0.213)
Self-rated health, reversed 1-7	-0.447***	-0.517***
	(0.083)	(0.083)
Log household income centered	-0.142	-0.165
	(0.097)	(0.097)
City: 1 Chengdu, 0 Beijing	0.071	0.263
	(0.137)	(0.137)
WIPV mean score, standardized		0.960***
		(0.108)
WIPV mean score, standardized # Social contact frequency, standardized		-0.276***
		(0.065)
Constant	5.876***	6.038***
	(0.497)	(0.501)
Observations	1702	1705
R2	0.159	0.186
Adjusted R2	0.153	0.181

Note: Unstandardized coefficients are reported with standard errors in parentheses unless otherwise indicated. * $p < .05$, ** $p < .01$, *** $p < .001$.

Table S9. Combined models and joint exposure analysis

	(1)	(2)	(3)	(4)
	**PHQ-9 depressive symptoms total score, complete 9 items, 0-27**	**PHQ-9 depressive symptoms total score, complete 9 items, 0-27**	**PHQ-9 depressive symptoms total score, complete 9 items, 0-27**	**PHQ-9 depressive symptoms total score, complete 9 items, 0-27**
WIPV mean score, standardized	0.704***	0.947***	1.064***	
	(0.185)	(0.193)	(0.194)	
PPA mean score, standardized	0.120	0.189	0.122	
	(0.164)	(0.164)	(0.157)	
Social contact frequency, standardized	-0.153	0.022	0.027	0.004
	(0.099)	(0.093)	(0.091)	(0.097)
Age centered	0.002	0.005	0.005	0.003
	(0.009)	(0.008)	(0.009)	(0.009)
Urban/non-agricultural hukou	-0.760***	-0.668**	-0.703**	-0.908***
	(0.216)	(0.217)	(0.215)	(0.217)
Ethnic minority	4.208***	4.274***	4.222***	4.431***
	(0.841)	(0.809)	(0.806)	(0.843)
CCP member	1.243***	1.483***	1.495***	1.598***
	(0.357)	(0.352)	(0.352)	(0.348)
Education centered	-0.061	-0.056	-0.053	-0.057
	(0.032)	(0.030)	(0.030)	(0.032)
Currently married or remarried	-0.492	-0.385	-0.367	-0.415
	(0.264)	(0.273)	(0.276)	(0.271)
Self-rated health, reversed 1-7	-0.523***	-0.446***	-0.475***	-0.518***
	(0.111)	(0.110)	(0.111)	(0.111)
Log household income centered	0.055	0.039	0.031	0.042
	(0.140)	(0.139)	(0.140)	(0.140)
City: 1 Chengdu, 0 Beijing	0.098	0.057	0.055	-0.040
	(0.164)	(0.166)	(0.165)	(0.161)
PPA mean score, standardized # Social contact frequency, standardized		-0.533***		
		(0.083)		
WIPV mean score, standardized # Social contact frequency, standardized			-0.487***	
			(0.082)	
No PPA or WIPV				0.000
				(.)
PPA only				0.334
				(0.184)
WIPV only				1.180*
				(0.570)
Both PPA and WIPV				1.459***
				(0.217)
Constant	6.916***	6.419***	6.582***	6.364***
	(0.636)	(0.639)	(0.644)	(0.650)
Observations	1849	1849	1849	1849
R2	0.166	0.198	0.196	0.159
Adjusted R2	0.161	0.193	0.190	0.153

Note: Unstandardized coefficients are reported with standard errors in parentheses unless otherwise indicated. * $p < .05$, ** $p < .01$, *** $p < .001$.

Table S10. Sensitivity models excluding self-rated health

	(1)	(2)	(3)	(4)
	**PHQ-9 depressive symptoms total score, complete 9 items, 0-27**	**PHQ-9 depressive symptoms total score, complete 9 items, 0-27**	**PHQ-9 depressive symptoms total score, complete 9 items, 0-27**	**PHQ-9 depressive symptoms total score, complete 9 items, 0-27**
PPA mean score, standardized	0.616***		0.814***	
	(0.114)		(0.116)	
Social contact frequency, standardized	-0.181	-0.243*	0.006	-0.048
	(0.101)	(0.101)	(0.093)	(0.092)
Age centered	0.017*	0.016*	0.019*	0.017*
	(0.008)	(0.008)	(0.008)	(0.008)
Urban/non-agricultural hukou	-0.915***	-0.756***	-0.891***	-0.698**
	(0.216)	(0.218)	(0.216)	(0.216)
Ethnic minority	4.328***	4.084***	4.482***	4.110***
	(0.860)	(0.838)	(0.841)	(0.805)
CCP member	1.369***	1.156**	1.664***	1.426***
	(0.363)	(0.360)	(0.362)	(0.354)
Education centered	-0.069*	-0.063	-0.067*	-0.055
	(0.032)	(0.032)	(0.030)	(0.031)
Currently married or remarried	-0.517	-0.521*	-0.421	-0.389
	(0.270)	(0.265)	(0.279)	(0.277)
Log household income centered	0.030	0.026	0.017	0.003
	(0.139)	(0.141)	(0.138)	(0.141)
City: 1 Chengdu, 0 Beijing	-0.101	0.150	-0.211	0.103
	(0.159)	(0.160)	(0.161)	(0.161)
WIPV mean score, standardized		0.803***		1.178***
		(0.132)		(0.149)
PPA mean score, standardized # Social contact frequency, standardized			-0.469***	
			(0.078)	
WIPV mean score, standardized # Social contact frequency, standardized				-0.505***
				(0.083)
Constant	4.419***	4.234***	4.391***	4.145***
	(0.304)	(0.299)	(0.316)	(0.312)
Observations	1849	1849	1849	1849
R2	0.143	0.153	0.169	0.184
Adjusted R2	0.138	0.148	0.164	0.179

Note: Unstandardized coefficients are reported with standard errors in parentheses unless otherwise indicated. * p < .05, ** p < .01, *** p < .001.

Table S11. Alternative PHQ-9 outcome models

	(1)	(2)	(3)	(4)	(5)	(6)
	**PHQ-9 depressive symptoms total score, complete 9 items, 0-27**	**PHQ-9 depressive symptoms total score, complete 9 items, 0-27**	**PHQ-9 >= 5**	**PHQ-9 >= 5**	**PHQ-9 >= 10**	**PHQ-9 >= 10**
main						
PPA mean score, standardized	0.163***		0.384***		0.578***	
	(0.025)		(0.063)		(0.098)	
Social contact frequency, standardized	0.031	0.023	0.181**	0.158*	0.233*	0.193
	(0.026)	(0.026)	(0.062)	(0.062)	(0.100)	(0.101)
PPA mean score, standardized # Social contact frequency, standardized	-0.089***		-0.251***		-0.319***	
	(0.017)		(0.043)		(0.058)	
Age centered	0.002	0.002	0.003	0.002	-0.003	-0.005
	(0.002)	(0.002)	(0.005)	(0.005)	(0.009)	(0.009)
Urban/non-agricultural hukou	-0.253***	-0.217***	-0.451**	-0.364*	-0.685**	-0.612**
	(0.056)	(0.056)	(0.137)	(0.142)	(0.223)	(0.228)
Ethnic minority	0.821***	0.779***	1.898***	1.715***	2.251***	2.077***
	(0.118)	(0.106)	(0.348)	(0.344)	(0.439)	(0.412)
CCP member	0.407***	0.345***	0.837***	0.745***	1.067***	0.895**
	(0.075)	(0.076)	(0.168)	(0.172)	(0.277)	(0.283)
Education centered	-0.016*	-0.014*	-0.026	-0.020	-0.041	-0.041
	(0.007)	(0.007)	(0.017)	(0.017)	(0.028)	(0.028)
Currently married or remarried	-0.097	-0.104	-0.384*	-0.365*	0.123	0.105
	(0.069)	(0.066)	(0.171)	(0.171)	(0.325)	(0.318)
Self-rated health, reversed 1-7	-0.132***	-0.146***	-0.254***	-0.288***	-0.276*	-0.337*
	(0.030)	(0.031)	(0.067)	(0.068)	(0.132)	(0.132)
Log household income centered	0.007	0.006	0.033	0.027	0.181	0.176
	(0.031)	(0.031)	(0.072)	(0.075)	(0.121)	(0.126)
City: 1 Chengdu, 0 Beijing	-0.042	0.030	-0.220	-0.063	-0.904***	-0.662*
	(0.050)	(0.051)	(0.126)	(0.129)	(0.254)	(0.268)
WIPV mean score, standardized		0.227***		0.540***		0.678***
		(0.026)		(0.079)		(0.107)
WIPV mean score, standardized # Social contact frequency, standardized		-0.094***		-0.253***		-0.291***
		(0.017)		(0.045)		(0.061)
Constant	2.096***	2.122***	0.924*	0.973*	-0.951	-0.742
	(0.167)	(0.170)	(0.386)	(0.394)	(0.735)	(0.754)
Observations	1849	1849	1849	1849	1849	1849

Note: Models 1–2 use the PHQ-9 total score (0–27) as the outcome and are estimated using Poisson regression with robust standard errors. Models 3–4 use PHQ-9 ≥ 5 and Models 5–6 use PHQ-9 ≥ 10 as binary outcomes and are estimated using logistic regression with robust standard errors. Odd-numbered models estimate the PPA × social contact frequency interaction, whereas even-numbered models estimate the WIPV × social contact frequency interaction. All models adjust for the full set of covariates. Coefficients are reported with robust standard errors in parentheses. p < .05, p < .01, p < .001.

Table S12. Table 5 models additionally adjusted for parental education at age 12

	(1)	(2)	(3)
	**PHQ-9 depressive symptoms total score, complete 9 items, 0-27**	**PHQ-9 depressive symptoms total score, complete 9 items, 0-27**	**PHQ-9 depressive symptoms total score, complete 9 items, 0-27**
WIPV mean score, standardized	0.789***	0.989***	1.124***
	(0.192)	(0.198)	(0.195)
PPA mean score, standardized	0.077	0.176	0.087
	(0.167)	(0.168)	(0.160)
Social contact frequency, standardized	-0.123	0.036	0.050
	(0.103)	(0.097)	(0.095)
Age centered	0.014	0.017	0.017
	(0.010)	(0.010)	(0.010)
Urban/non-agricultural hukou	-0.789***	-0.694**	-0.752***
	(0.231)	(0.231)	(0.228)
Ethnic minority	4.226***	4.261***	4.228***
	(0.859)	(0.831)	(0.827)
CCP member	0.991**	1.215***	1.252***
	(0.373)	(0.368)	(0.369)
Education centered	-0.085*	-0.084**	-0.079*
	(0.034)	(0.033)	(0.032)
Currently married or remarried	-0.735**	-0.606*	-0.618*
	(0.273)	(0.284)	(0.285)
Self-rated health, reversed 1-7	-0.570***	-0.494***	-0.523***
	(0.116)	(0.115)	(0.115)
Log household income centered	0.127	0.103	0.107
	(0.146)	(0.146)	(0.147)
Father's education at age 12 centered	0.022	0.014	0.016
	(0.035)	(0.035)	(0.035)
Mother's education at age 12 centered	0.075*	0.085**	0.082*
	(0.034)	(0.033)	(0.033)
City: 1 Chengdu, 0 Beijing	0.377*	0.333	0.338
	(0.174)	(0.176)	(0.175)
PPA mean score, standardized # Social contact frequency, standardized		-0.499***	
		(0.089)	
WIPV mean score, standardized # Social contact frequency, standardized			-0.475***
			(0.087)
Constant	7.277***	6.769***	6.969***
	(0.671)	(0.672)	(0.674)
Observations	1732	1732	1732
R2	0.174	0.202	0.202
Adjusted R2	0.167	0.195	0.195

Note: Unstandardized coefficients are reported with standard errors in parentheses. All models additionally adjust for paternal and maternal education when the respondent was age 12, entered separately as centered years of education. The PPA and WIPV interactions are estimated in separate models. * $p < .05$, ** $p < .01$, *** $p < .001$.

Table S13. Additional Robustness and Diagnostic Checks

Panel A. Sensitivity to the minimum number of valid social-contact items

Minimum valid items	PPA × contact b [95% CI]	N	WIPV × contact b [95% CI]	N
1	−.442*** [−.595, −.289]	1849	−.487*** [−.648, −.326]	1849
3	−.411*** [−.564, −.257]	1829	−.465*** [−.627, −.302]	1829
5	−.439*** [−.598, −.279]	1802	−.461*** [−.626, −.296]	1802
7	−.547*** [−.735, −.359]	1320	−.527*** [−.719, −.335]	1320

Panel B. Observed violence-score ranges within social-contact quintiles

Social-contact quintile	N	PPA observed range	WIPV observed range
Q1	393	0.0 to 4.0	0.0 to 4.0
Q2	414	0.0 to 4.0	0.0 to 4.0
Q3	406	0.0 to 4.0	0.0 to 4.0
Q4	311	0.0 to 4.0	0.0 to 4.0
Q5	325	0.0 to 4.0	0.0 to 4.0

Panel C. Breusch-Pagan/Cook-Weisberg tests for heteroskedasticity

Primary model	χ^2	p
Model 1 Baseline	168.58	< .001
Model 2 PPA	212.12	< .001
Model 3 WIPV	264.13	< .001
Model 4 PPA interaction	180.28	< .001
Model 5 WIPV interaction	229.78	< .001

Panel D. Cook's distance diagnostics

Interaction model	N	4/N threshold	Maximum Cook's D
Model 4 PPA interaction	1849	0.0022	0.055
Model 5 WIPV interaction	1849	0.0022	0.052

Note: Panel A reports separate PPA and WIPV interaction models using the same standardized focal variables and covariate set as the primary models. Panel B is descriptive. Panel C uses the non-robust versions of Models 1-5 solely for the heteroskedasticity test; all primary estimates use HC1 robust inference. Panel D reports influence diagnostics from the corresponding non-robust OLS interaction specifications. * $p < .05$, ** $p < .01$, *** $p < .001$.

Table S14. City-Specific Moderation Analyses

Panel A. Separate-city interaction estimates

Exposure interaction	Beijing b [95% CI]	N	Chengdu b [95% CI]	N
PPA	−.686*** [−.875, −.496]	1075	−.214* [−.414, −.014]	774
WIPV	−.697*** [−.892, −.502]	1075	−.451** [−.753, −.148]	774

Panel B. Formal city-difference tests from pooled three-way interaction models

Exposure	Chengdu − Beijing difference b [95% CI]	p	N
PPA	.541*** [.265, .817]	$< .001$	1849
WIPV	.302 [−.069, .674]	$= .111$	1849

Note: Panel A reports separate city-specific OLS models. Panel B reports the city × exposure × social-contact coefficient from pooled models containing all lower-order terms. All models adjust for age, urban hukou, ethnic minority status, CCP membership, education, marital status, self-rated health, and household income. PPA, WIPV, and social contact frequency were standardized in the full analytic sample. * $p < .05$, ** $p < .01$, *** $p < .001$.

Supplementary Figures

Figure S1. Johnson–Neyman-style probing of the PPA × social contact frequency interaction.

The plot shows the conditional slope of PPA across the observed range of standardized social contact frequency, with 95% confidence intervals.

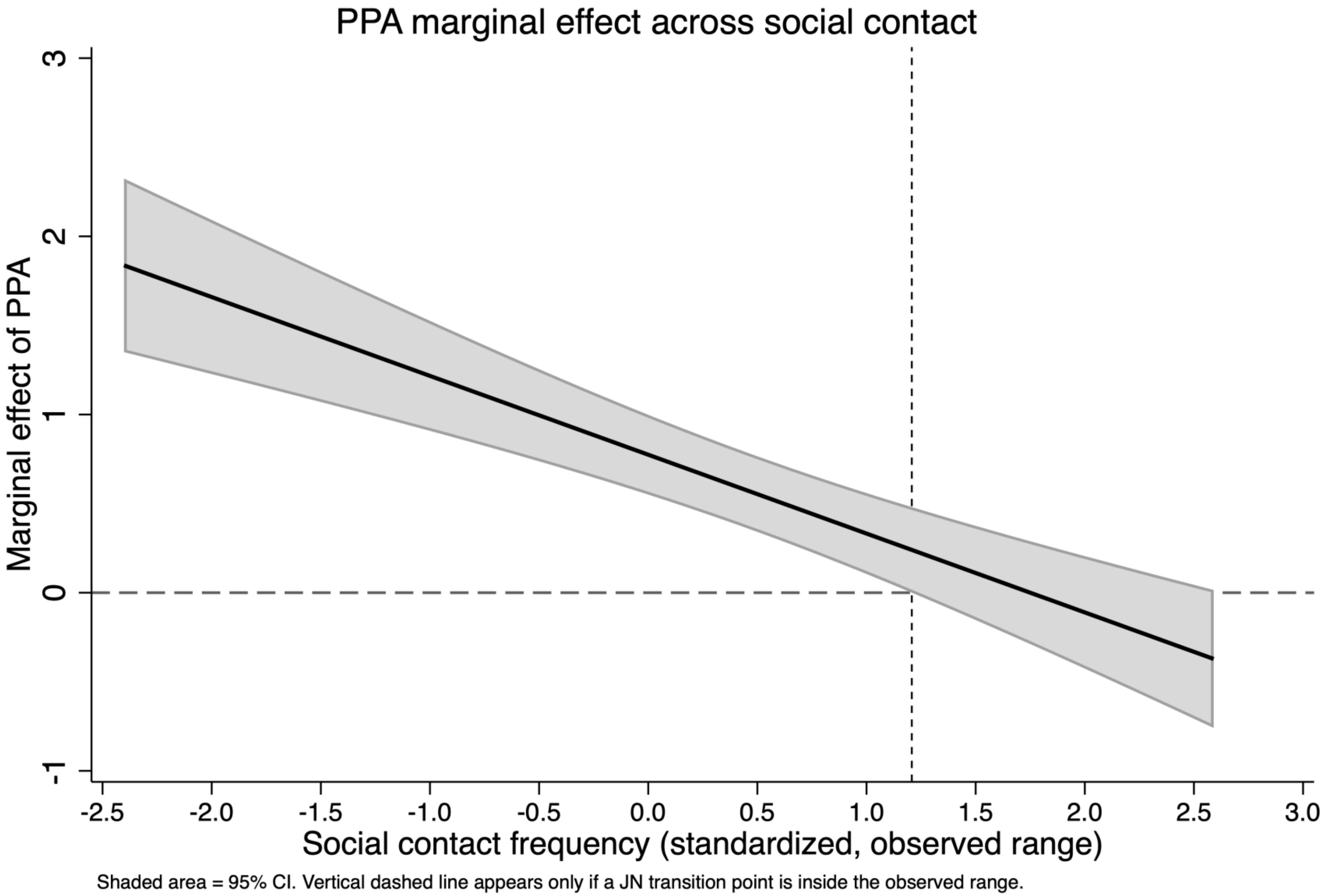

Figure S2. Johnson–Neyman-style probing of the WIPV × social contact frequency interaction.

The plot shows the conditional slope of WIPV across the observed range of standardized social contact frequency, with 95% confidence intervals.

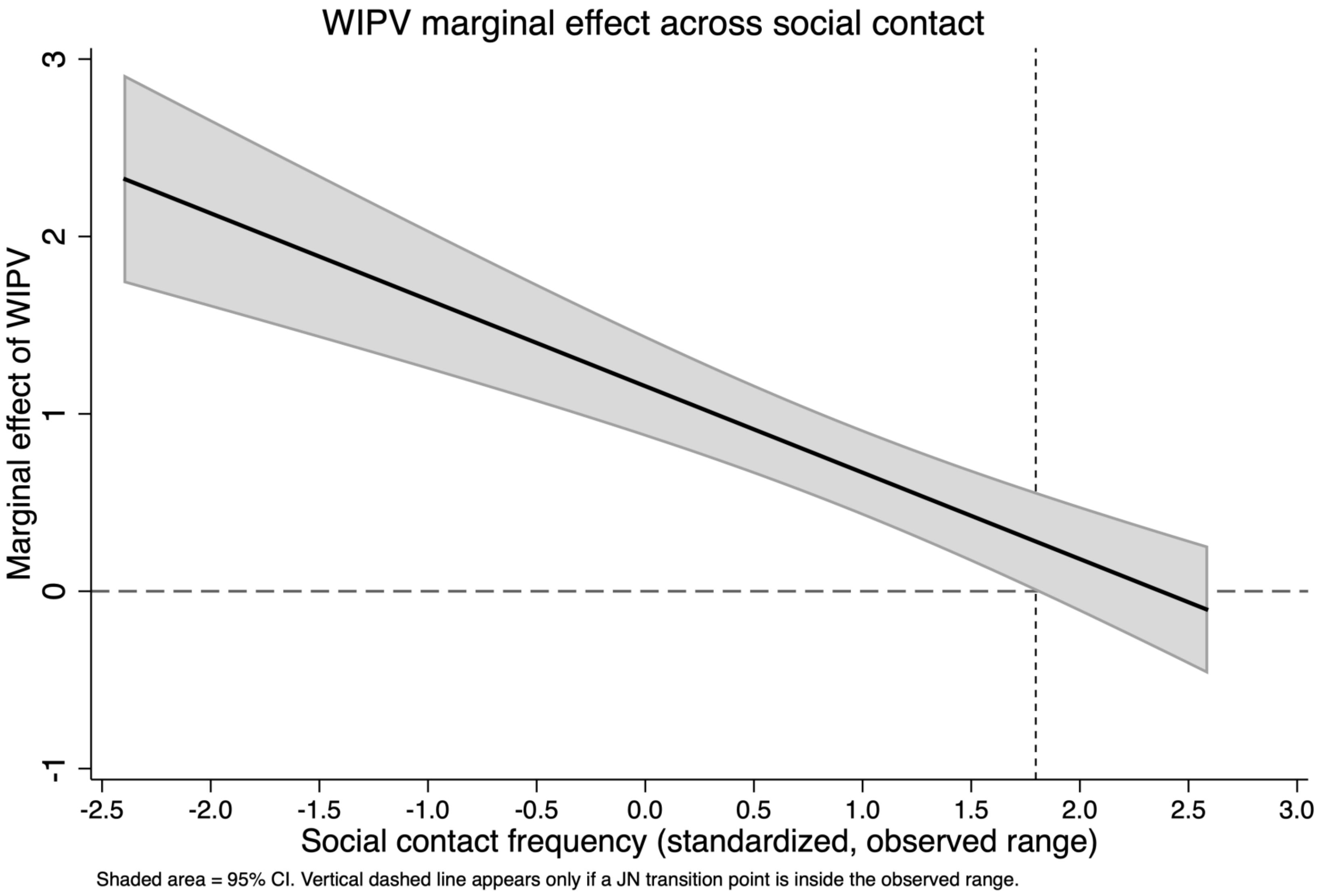

Figure S3. Distribution of PPA and WIPV exposure scores.

This figure describes the observed exposure distribution and helps evaluate whether results are driven by a small number of highly exposed respondents.

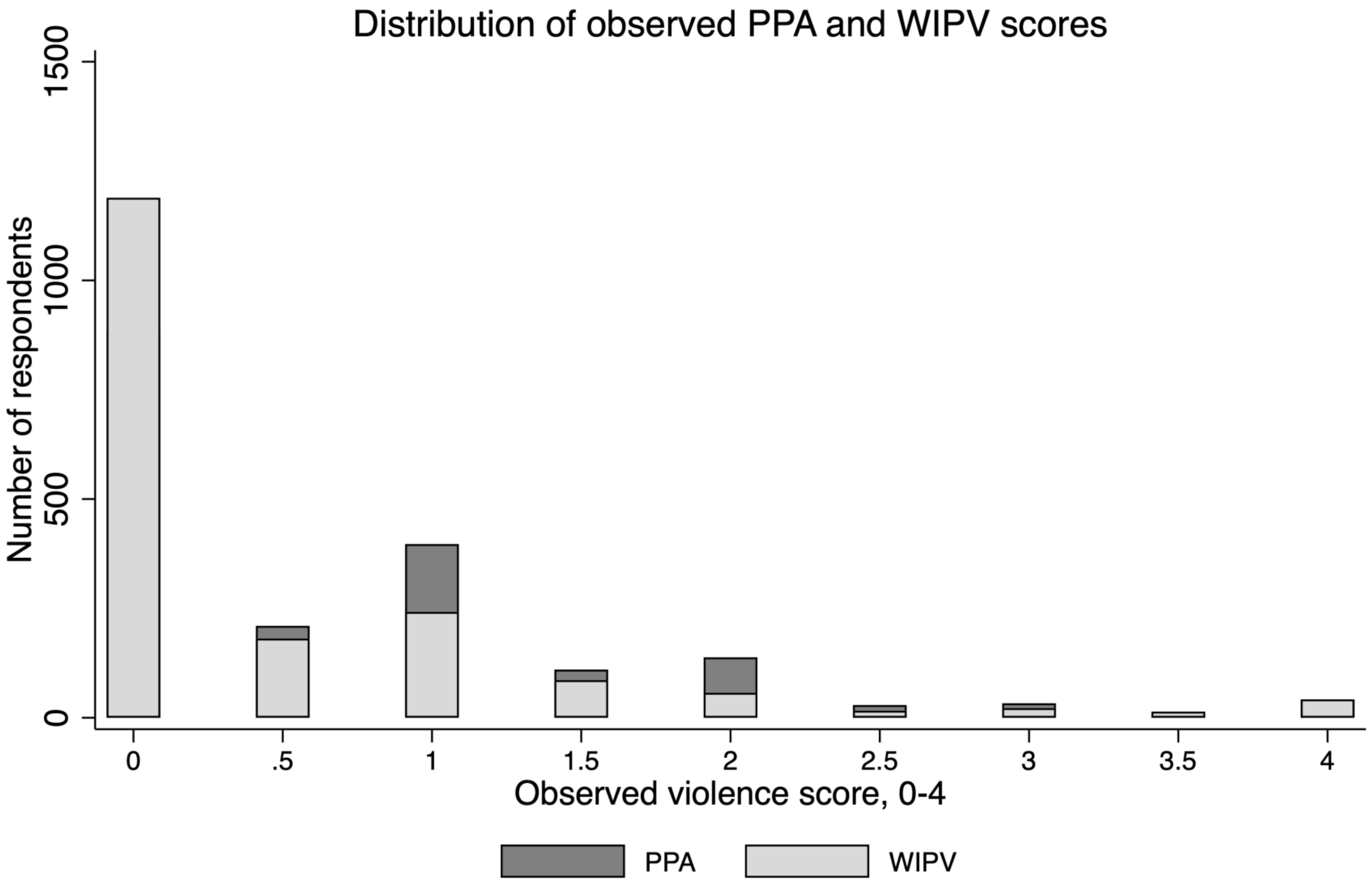

Observed values are concentrated at low exposure levels; high-exposure cells are shown explicitly.

Figure S4. Cook's distance by PPA exposure level.

This diagnostic figure evaluates influential observations across the observed PPA exposure distribution.

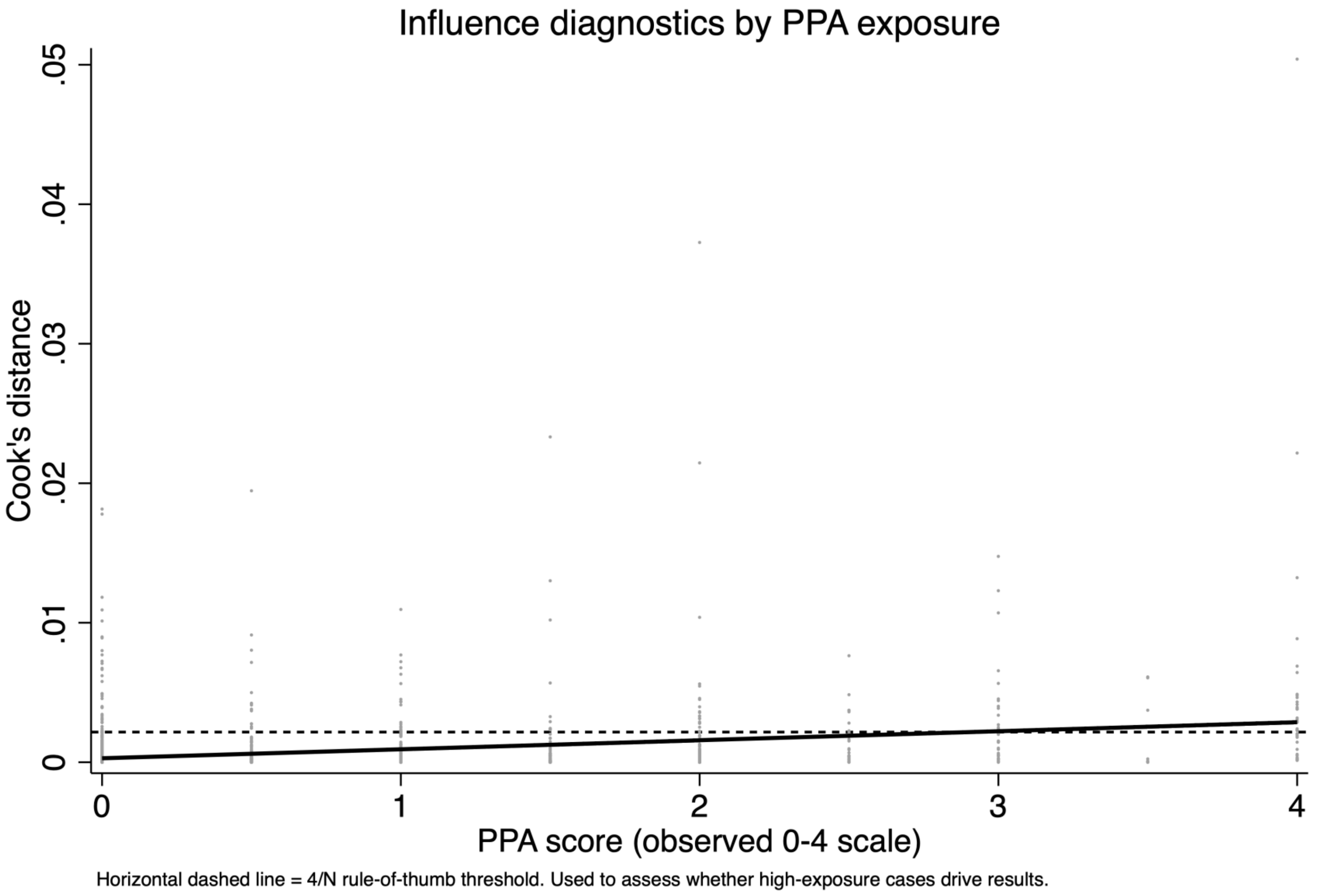

Figure S5. Cook's distance by WIPV exposure level.

This diagnostic figure evaluates influential observations across the observed WIPV exposure distribution.

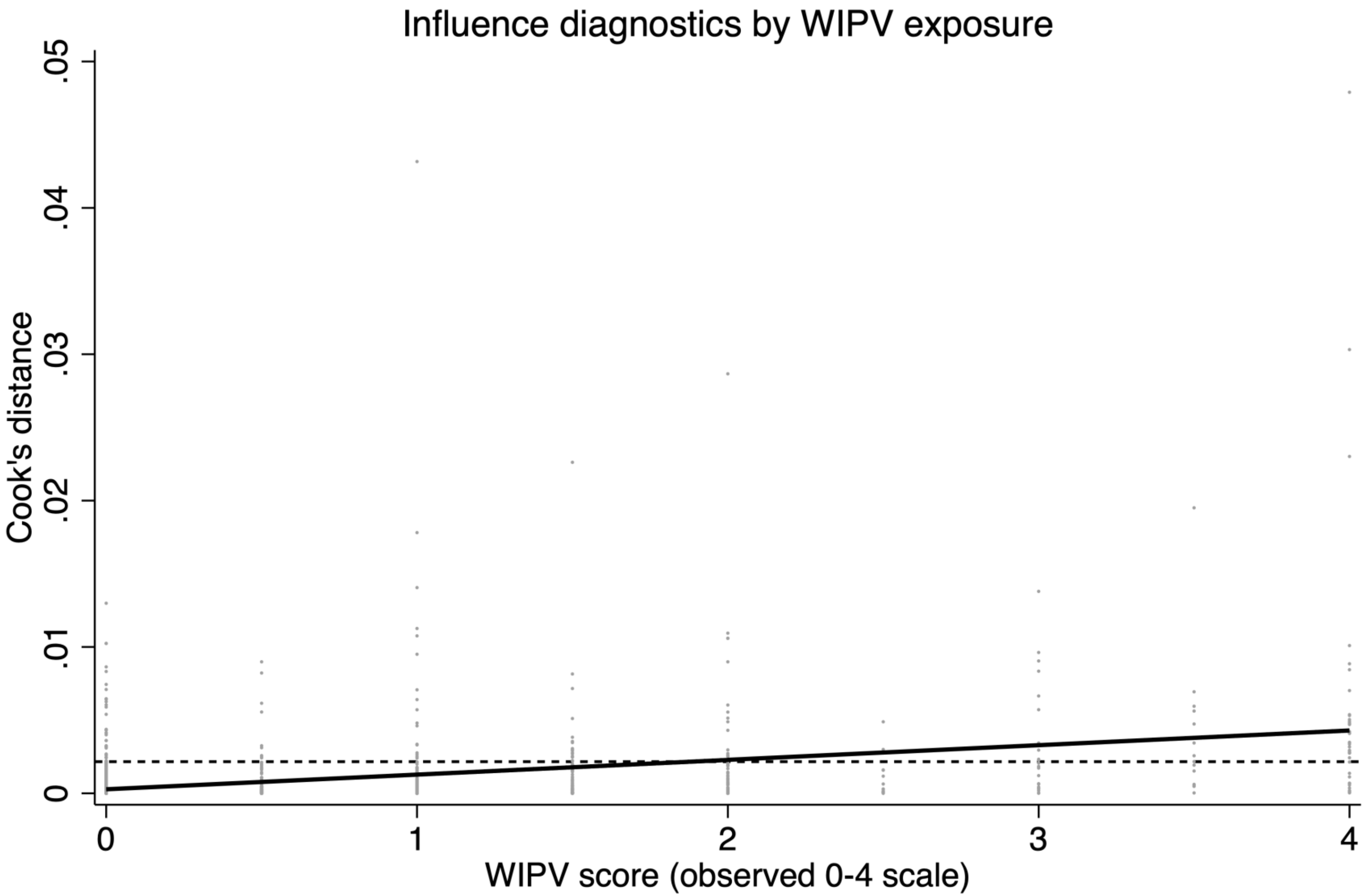

Horizontal dashed line = 4/N rule-of-thumb threshold. Used to assess whether high-exposure cases drive results.

Figure S6. Residual-versus-fitted diagnostic plot for the PPA moderation model.

This figure is an OLS diagnostic plot and should be interpreted as model-checking evidence rather than substantive evidence of moderation.

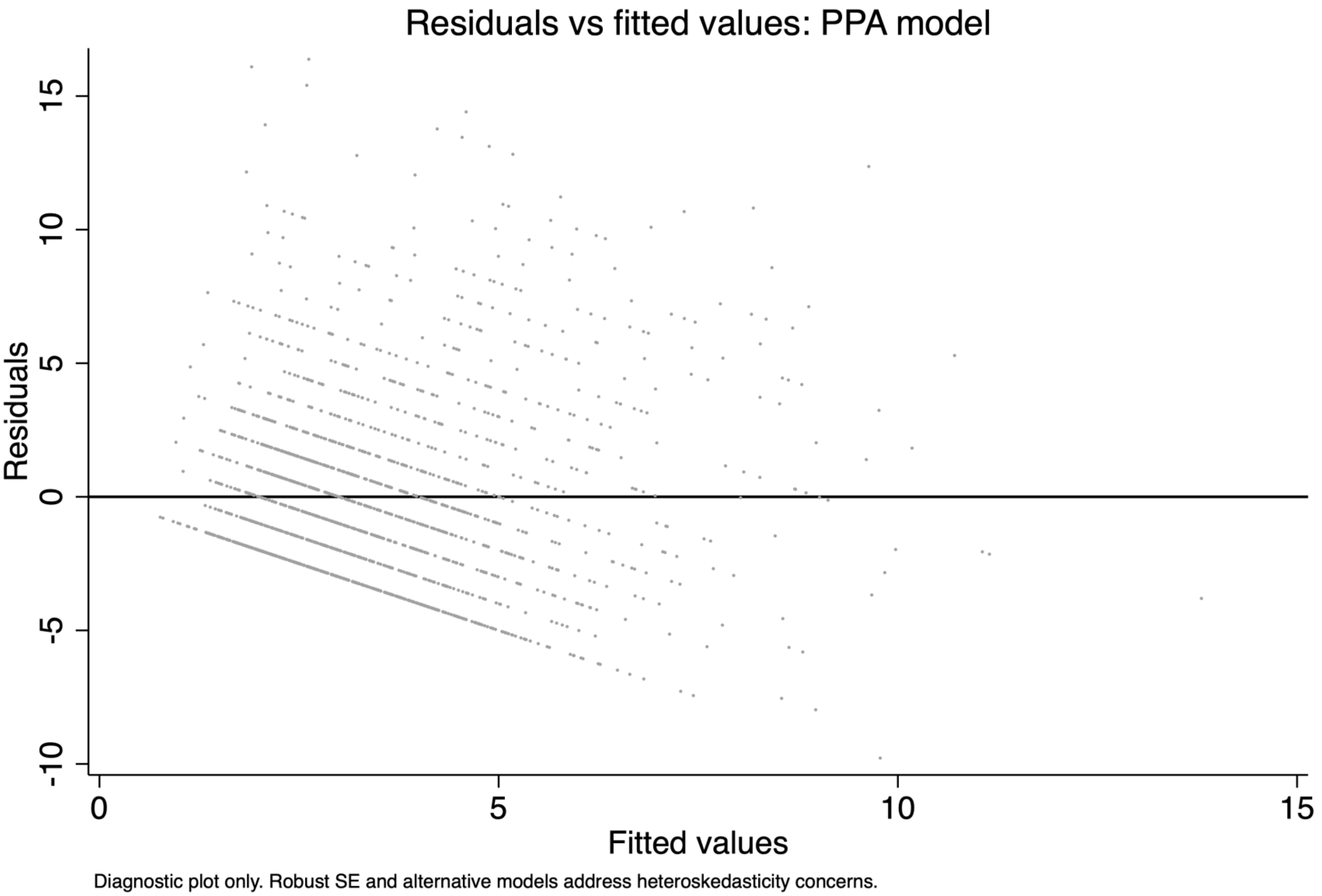

Figure S7. Residual-versus-fitted diagnostic plot for the WIPV moderation model.

This figure is an OLS diagnostic plot and should be interpreted as model-checking evidence rather than substantive evidence of moderation.

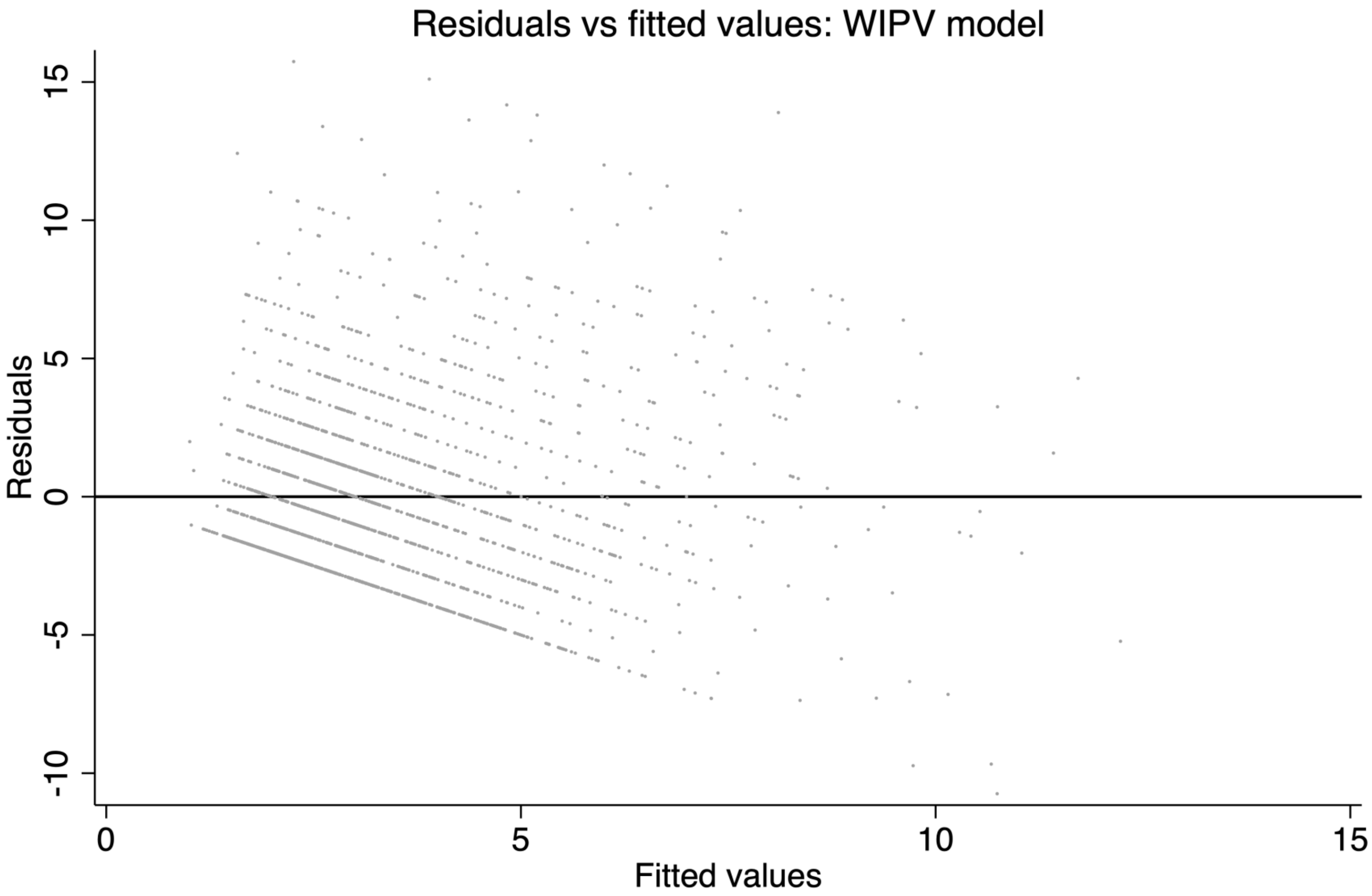

Diagnostic plot only. Robust SE and alternative models address heteroskedasticity concerns.
